# Comparison of Fecal Collection Methods on Variation in Gut Metagenomics and Untargeted Metabolomics

**DOI:** 10.1128/mSphere.00636-21

**Published:** 2021-09-15

**Authors:** Huihui Guan, Yanni Pu, Chenglin Liu, Tao Lou, Shishang Tan, Mengmeng Kong, Zhonghan Sun, Zhendong Mei, Qibin Qi, Zhexue Quan, Guoping Zhao, Yan Zheng

**Affiliations:** a Human Phenome Institute, School of Life Sciences, Fudan University, Shanghai, China; b MOE Key Laboratory of Contemporary Anthropology, School of Life Sciences, Fudan University, Shanghai, China; c Department of Epidemiology and Population Health, Albert Einstein College of Medicine, Bronx, New York, USA; d Ministry of Education Key Laboratory for Biodiversity Science and Ecological Engineering, Institute of Biodiversity Science, School of Life Sciences, Fudan University, Shanghai, China; e Institute of Plant Physiology and Ecology, Shanghai Institutes for Biological Sciences, Chinese Academy of Sciences, Shanghai, China; f State Key Laboratory of Genetic Engineering School of Life Sciences, Human Phenome Institute, Fudan University, Shanghai, China; g Ministry of Education Key Laboratory of Public Health Safety, School of Public Health, Fudan University, Shanghai, China; h Yiwu Research Institute of Fudan University, Shanghai, China; University of California, Davis

**Keywords:** collection method, feces, metabolomics, metagenomics, reliability, whole-genome shotgun sequencing

## Abstract

Integrative analysis of high-quality metagenomics and metabolomics data from fecal samples provides novel clues for the mechanism underpinning gut microbe-human interactions. However, data regarding the influence of fecal collection methods on both metagenomics and metabolomics are sparse. Six fecal collection methods (the gold standard [GS] [i.e., immediate freezing at −80°C with no solution], 95% ethanol, RNAlater, OMNIgene Gut, fecal occult blood test [FOBT] cards, and Microlution) were used to collect 88 fecal samples from eight healthy volunteers for whole-genome shotgun sequencing (WGSS) and untargeted metabolomic profiling. Metrics assessed included the abundances of predominant phyla and α- and β-diversity at the species, gene, and pathway levels. Intraclass correlation coefficients (ICCs) were calculated for microbes and metabolites to estimate (i) stability (day 4 versus day 0 within each method), (ii) concordance (day 0 for each method versus the GS), and (iii) reliability (day 4 for each method versus the GS). For the top 4 phyla and microbial diversity metrics at the species, gene, and pathway levels, generally high stability and reliability were observed for most methods except for 95% ethanol; similar concordances were seen for different methods. For metabolomics data, 95% ethanol showed the highest stability, concordance, and reliability (median ICCs = 0.71, 0.71, and 0.65, respectively). Taken together, OMNIgene Gut, FOBT cards, RNAlater, and Microlution, but not 95% ethanol, were reliable collection methods for gut metagenomic studies. However, 95% ethanol was the best for preserving fecal metabolite profiles. We recommend using separate collecting methods for gut metagenomic sequencing and fecal metabolomic profiling in large population studies.

**IMPORTANCE** The choice of fecal collection method is essential for studying gut microbe-human interactions in large-scale population-based research. In this study, we examined the effects of fecal collection methods and storage time at ambient temperature on variations in the gut microbiome community composition; microbial diversity metrics at the species, gene, and pathway levels; antibiotic resistance genes; and metabolome profiling. Our findings suggest using different fecal sample collection methods for different data generation purposes. OMNIgene Gut, FOBT cards, RNAlater, and Microlution, but not 95% ethanol, were reliable collection methods for gut metagenomic studies. However, 95% ethanol was the best for preserving fecal metabolite profiles.

## INTRODUCTION

In recent years, growing evidence suggests that gut microbiota dysbiosis may be an essential factor in the pathogenesis of various diseases such as gastrointestinal diseases (1–3), cardiometabolic diseases (4, 5), neurodegenerative disease (6), and hepatic illnesses (7, 8). The integration of high-throughput multi-omics data of gut microbiota, which are usually metagenomics and metabolomics data in population-based studies (9–12), facilitates a better understanding of molecular function. Nowadays, increasing numbers of large-scale epidemiological studies have collected fecal samples to evaluate the role of the gut microbiome in disease etiology. Immediate freezing at −80°C with no solution has been considered the “gold standard” (GS) for fecal sample collection, as it preserved a microbial composition similar to that of a fresh sample and avoided the potential influence of added preservatives (13). However, this approach is impractical for field studies, especially for large-scale epidemiological studies, due to the unavailability of freezers and high shipping costs (14). An ideal on-site fecal sampling method should be able to keep biomarkers as consistent with those of the GS as possible (e.g., DNA for 16S rRNA amplicon sequencing and metagenomics and small molecules for metabolomics), be stable under ambient temperature for multiple days of transportation, and be appropriate for generating multi-omics data simultaneously.

Several fecal collection methods such as RNAlater and ethanol had been evaluated in previous microbiome studies, and most of these studies used 16S rRNA amplicon sequencing (13–17). For example, Song et al. compared five preservation methods for fecal specimens using 16S rRNA sequencing data. They found that 95% ethanol, Flinders Technology Associates (FTA) cards, and OMNIgene Gut preserved samples sufficiently well at ambient temperature (14). Thus far, two studies have investigated the stability and concordance of fecal samples using whole-genome shotgun sequencing (WGSS) (18, 19). Franzosa et al. (19) found that within-subject microbial species and genes were highly concordant using 95% ethanol and RNAlater. Byrd et al. (18) indicated that fecal occult blood test (FOBT) cards, fecal immunochemical test (FIT) tubes, and RNAlater but not 95% ethanol were acceptable choices as fecal sample collection methods in WGSS studies because of the excellent stability in preserving the microbial profiles of fecal samples. Only one study compared these methods for generating metabolomics data and suggested that 95% ethanol and FOBT cards, but not FIT tubes, were good options for keeping high stability of metabolites (13). In addition, there has been only one study that integrated 16S rRNA sequencing and metabolomics using fecal samples within one design (20), which found that FTA cards demonstrated the highest concordance with the GS for α-diversity, followed by OMNIgene Gut and RNAlater, while 95% ethanol detected the most similar metabolites to the GS. Thus far, it is unclear whether one single collection method would be recommended for fecal samples based on WGSS and metabolomics simultaneously. Meanwhile, no integration study of metagenomics and metabolomics has yet estimated the influence of sample collection methods and storage time at ambient temperature on functional bacteria, microbial metabolites, and antibiotic resistance genes.

In metagenomics and metabolomics studies, functional microbial metabolites and antibiotic resistance genes are important research objects. It has been suggested that the gut microbiota plays a critical role in human health and diseases through microbial metabolites such as short-chain fatty acids (SCFAs) and secondary bile acids (SBAs), which serve as signaling molecules or energy substrates (21–24). Besides, antibiotic resistance genes of human pathogens can impact the clinical outcomes of human disease by enhancing virulence and limiting available treatments (25) and have become one of the greatest threats to global health (26).

To better understand the impact of the fecal collection method on variations in the microbiome and metabolome, we compared six fecal sample collection methods (GS, 95% ethanol, RNAlater, OMNIgene Gut, FOBT cards, and Microlution) using WGSS and mass spectrometry (MS)-based untargeted metabolomics profiles. We designed delayed freezing of samples stored at ambient temperature for 4 days after collection to simulate the effect of carrier transport. We evaluated the performance of each collection method in preserving the microbiome and metabolome through 3 measurements: (i) stability (i.e., comparison of samples frozen after 4 days with those frozen immediately with the same collection method), (ii) concordance (i.e., comparison of samples frozen immediately for each method with those for the GS), and (iii) reliability (i.e., comparison of samples frozen after 4 days for each method with those for the GS). We evaluated the relative abundances of the top 4 phyla and 15 microbial diversity metrics, i.e., 5 metrics (3 α-diversity indices [observed richness, Shannon index, and Simpson index] and 2 β-diversity indices [the first component of principal-coordinate analysis {PCoA} {PC1} based on Bray-Curtis distance and that based on Jaccard distance]) at each level: the species, the gene, and the pathway. We also evaluated the relative abundances of functional species involved in SCFA production, genera involved in SBA metabolism, and antibiotic resistance genes. In addition, the influences of preservation and storage time on fecal metabolites were also assessed.

## RESULTS

### Self-collected stool samples provided metagenome and metabolome profiles.

**FIG 1:**
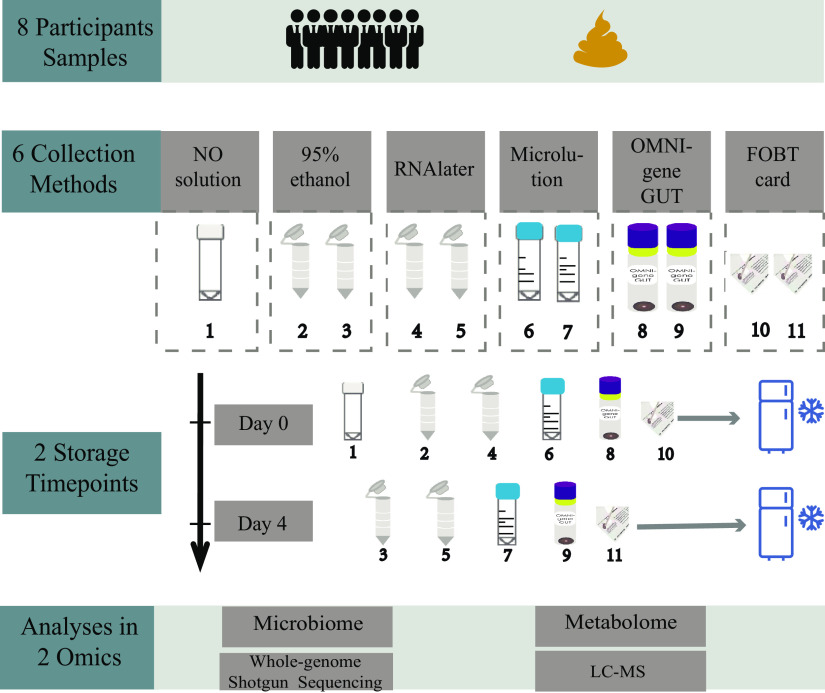
Flowchart of the study design. Samples stored on day 0 were frozen at −80°C soon after collection (∼30 min); samples stored on day 4 were frozen at −80°C after 4 days (96 h) at ambient temperature.

Eight self-reported healthy subjects (6 males and 2 females) aged 26.0 ± 1.5 years (mean ± standard deviation [SD]) were recruited from Fudan University, Shanghai, China, in August 2019 to provide stool samples in our study. Every individual collected 11 stool samples following the respective protocols by themselves (Fig. 1). Sample 1 was taken as the GS (i.e., frozen immediately with no solution) to represent the baseline microbiota composition. Samples 2 and 3 were preserved in 95% ethanol, samples 4 and 5 were preserved in RNAlater, samples 6 and 7 were preserved in Microlution, samples 8 and 9 were preserved in OMNIgene Gut, and samples 10 and 11 were preserved in FOBT cards. To evaluate the effects of different preservatives, samples 2, 4, 6, 8, and 10 were stored at −80°C within 30 min from collection (day 0) and then compared with GS samples in the analysis. To evaluate the stability of samples for each collection method over time, samples 3, 5, 7, 9, and 11 were stored at ambient temperature for 96 h (day 4) to mimic delays in freezing that often occur in the field due to the unavailability of refrigerators and carrier transport and then compared with samples 2, 4, 6, 8, and 10, respectively.

### Variation explained by individual, collection method, and storage time.

**FIG 2:**
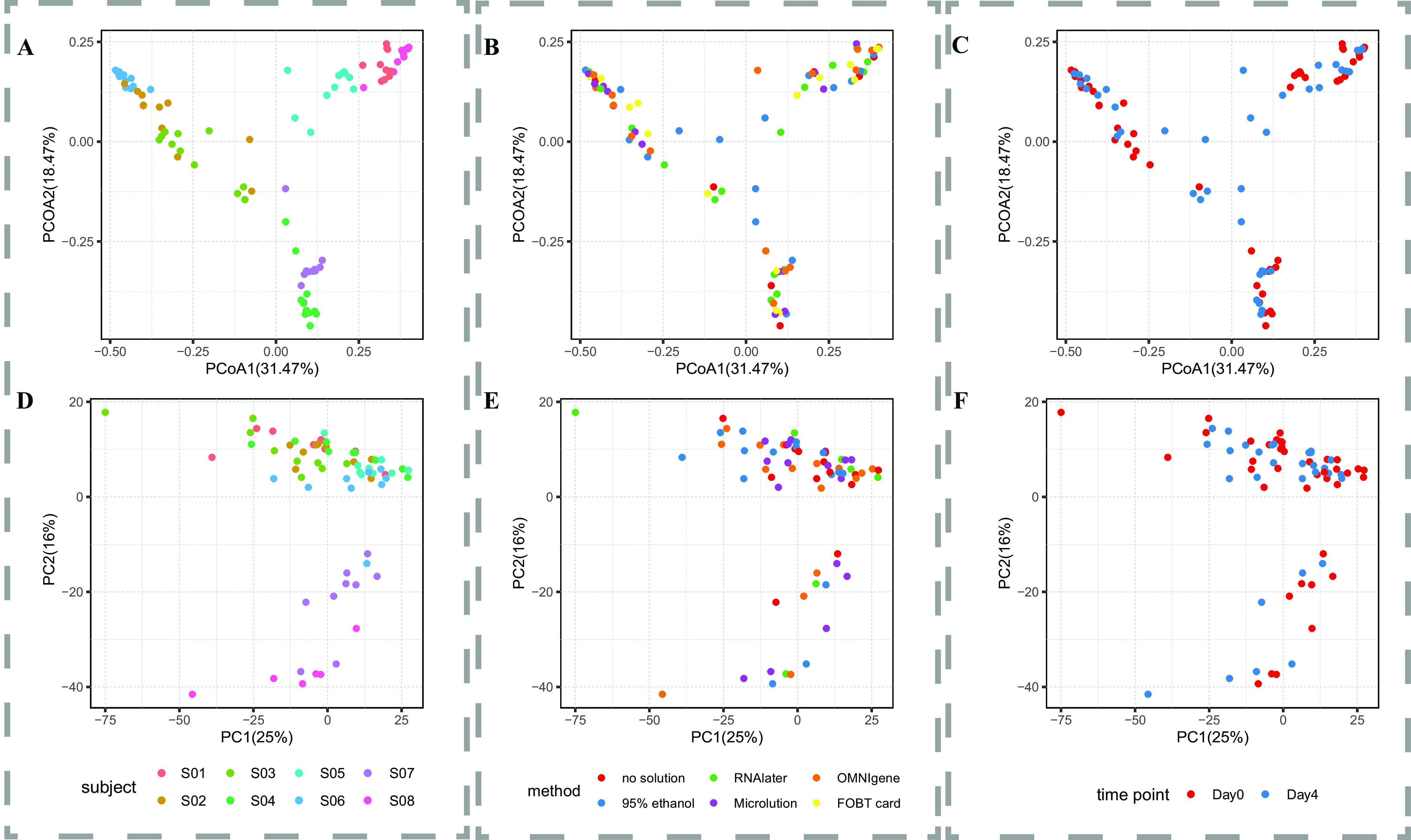
Clustering based on microbiota and metabolite variability. Shown are principal-coordinate analysis (PCoA) data based on the species profiles with different subjects (A), collection methods (B), and storage times at ambient temperature (C) and principal-component analysis (PCA) data based on metabolites with different subjects (D), collection methods (E), and storage times at ambient temperature (F).

The average dissimilarities among individuals were higher than those among collection methods at both the species level based on Bray-Curtis distance (see Fig. S1A and B in the supplemental material) and the metabolite level based on Euclidean distance (Fig. S1D and E), suggesting that the biological effects outweigh technical effects. The variations (Adonis) in species and metabolites were primarily explained by individuals and minimally explained by collection methods and storage times (Fig. 2). For example, interindividual variability explained 77% of the total variation in species and 37% of the variation in metabolites, whereas the collection method explained 3.6% and 4.9%, respectively, and the storage time explained 2.6% and 0.8%, respectively.

10.1128/mSphere.00636-21.2 FIG S1 Box plots showing the interindividual Bray-Curtis dissimilarities at the bacterial species level (A) or Euclidean distance at the metabolite level (C) based on the same method and same time point, the intermethod Bray-Curtis dissimilarities at the bacterial species level (B) or Euclidean distance at the metabolite level (D) based on the same individual and same time point, and inter-time-point Bray-Curtis dissimilarities at the bacterial species level (E) or Euclidean distance at the metabolite level (F) based on the same individual and same method. Download FIG S1, PDF file, 0.9 MB. Copyright © 2021 Guan et al. 2021 Guan et al. https://creativecommons.org/licenses/by/4.0/ This content is distributed under the terms of the Creative Commons Attribution 4.0 International license.

### Microbiome analyses. (i) Stability at ambient temperature.

When comparing fecal samples frozen on day 4 with those frozen on day 0 with the same collection method, we observed good stability as measured by intraclass correlation coefficients (ICCs) (<0.4 as poor, 0.4 to 0.75 as fair to good, and ≥0.75 as excellent) when using most preservation methods, except for 95% ethanol (Fig. S2A). Specifically, OMNIgene Gut showed the highest stability (most ICCs were ≥0.75 [*P* < 0.05]). The stability of the top 4 phyla and microbial diversity at the gene level varied largely among collection methods. Ninety-five-percent ethanol had low stability for two α-diversity measures (ICCs = 0.28 for Shannon and 0.20 for Simpson) at the species level, and all methods showed relatively high stability (all ICCs were >0.84) for two β-diversity measures (Bray-Curtis distance PC1 and Jaccard distance PC1) at the species level. OMNIgene Gut, FOBT cards, and RNAlater had high stability (all ICCs were >0.74) for microbial diversity at the pathway level (Fig. S2A). When comparing fecal samples frozen on day 4 with those frozen on day 0, significant differences were observed in the relative abundances of the top 4 phyla in fecal samples collected with 95% ethanol and in those of *Bacteroidetes* and *Actinobacteria* in samples collected with RNAlater (*P* < 0.05 by two-sided Wilcoxon rank sum tests) (Data Set S1, tab 1). No significant differences were observed in the top 4 phyla in fecal samples collected with Microlution, OMNIgene Gut, or FOBT cards in such comparisons (Data Set S1, tab 1). The intramethod performance using Bray-Curtis dissimilarity suggested high consistency in the relative abundances of species for most methods except for 95% ethanol (Fig. S2B). Stability for the top 20 most abundant pathways in samples collected using OMNIgene Gut, FOBT cards, and RNAlater was primarily superior to those for 95% ethanol or Microlution (Fig. S2C and E). For example, significant differences were observed in 13 pathways in fecal samples frozen on day 4 compared with those that were frozen on day 0 when collected with 95% ethanol (*P* < 0.05 by two-sided Wilcoxon rank sum tests) (Data Set S1, tab 1). The stability of preserving the top 20 most abundant resistance genes was generally high with most methods except for 95% ethanol (Fig. S2D and F). Two-sided Wilcoxon rank sum tests showed significant differences in 4 resistance genes (Comprehensive Antibiotic Resistance Database [CARD] accession numbers *ARO:3000191* [*tetQ*], *ARO:3000498* [ErmF], *ARO:3000518* [CRP], and *ARO:3000516* [*emrR*]) between samples frozen on day 0 and those frozen on day 4 when collected with 95% ethanol (*P* < 0.05) (Data Set S1, tab 1). When we extended the analysis to all detected pathways and resistance genes, the rank of collection methods in stability did not change (Fig. S2E and F).

10.1128/mSphere.00636-21.3 FIG S2 (A) Stability evaluation of four major phyla (red-underlined box), five microbial species metrics (green-underlined box), five gene diversity metrics (blue-underlined box), and five pathway diversity metrics (gray-underlined box). The asterisks on some bars represent a *P* value of <0.05 for the corresponding measurement indices for intraclass correlation coefficients (ICCs) using a two-sided test. (B) Intramethod β-diversity dissimilarities based on Bray-Curtis distance at the species level. (C to F) Stability evaluation of the top 20 most abundant pathways (C), the top 20 most abundant resistance genes (D), all pathways (E), and all resistance genes (F) using ICCs. Download FIG S2, PDF file, 1.97 MB. Copyright © 2021 Guan et al. 2021 Guan et al. https://creativecommons.org/licenses/by/4.0/ This content is distributed under the terms of the Creative Commons Attribution 4.0 International license.

10.1128/mSphere.00636-21.10 DATA SET S1 (Tab 1) *P* values of two-sided Wilcoxon rank sum tests on the top 4 phyla; 15 microbial diversity metrics at the species, gene, and pathway levels; and the top 20 most abundant pathways and resistance genes. (Tab 2) Multiple comparisons among different methods on day 0 for the top 4 phyla and 15 microbial diversity metrics at the species, gene, and pathway levels using a Kruskal-Wallis test. (Tab 3) Medians and interquartile ranges (IQRs) of the top 4 phyla and the top 20 most abundant pathways and resistance genes in samples collected with the gold standard and 95% ethanol (day 4) and their corresponding *P* values. (Tab 4) Multiple comparisons among different methods on day 4 for the top 4 phyla and 15 microbial diversity metrics at the species, gene, and pathway levels using a Kruskal-Wallis test. (Tab 5) Pairwise comparisons using *post hoc* Dunn’s multiple-comparison test between two collection methods following a statistically significant Kruskal-Wallis test (FDR-adjusted *P* value of <0.05) at the day 4 time point in *Actinobacteria*, *Bacteroidetes*, and *Firmicutes*. (Tab 6) Comparisons of medians (IQRs) across known metabolites using intraclass correlation coefficients (ICCs) and Spearman correlation coefficients (SCCs) with different collection methods at the ≥80% detectability level. (Tab 7) Comparisons of medians (IQRs) across known metabolites using ICCs for different collection methods at the ≥80% and 100% detectability levels. (Tab 8) Detectability of metabolites related to short-chain fatty acids and secondary bile acids for different fecal sample collection methods. Download Data Set S1, XLS file, 0.68 MB. Copyright © 2021 Guan et al. 2021 Guan et al. https://creativecommons.org/licenses/by/4.0/ This content is distributed under the terms of the Creative Commons Attribution 4.0 International license.

### (ii) Concordance compared to the GS on day 0.

When comparing the samples collected on day 0 using different methods with GS samples frozen immediately with no solution, the ICCs were generally low for the relative abundances of the top 4 phyla. All collection methods had similar concordance performances, in which ICCs for microbial diversity at the species level were generally higher (most ICCs were ≥0.75) than the ICCs for microbial diversity at the gene and pathway levels (all ICCs for Shannon and Simpson indices were <0.75) (Fig. S3A). Two-sided Wilcoxon rank sum tests showed no significant difference between samples collected with any method frozen on day 0 and GS samples (all *P* > 0.05) (Data Set S1, tab 1) on phylum abundance and diversity measures. The Bray-Curtis dissimilarities in the gut microbiome between 95% ethanol freezing on day 0 and the GS were similar to those between other methods and the GS (Fig. S3B). There were no significant differences in major phylum abundances and overall diversity indices at the species, gene, and pathway levels among all collection methods on day 0 (false discovery rate [FDR]-adjusted *P* value of >0.05 by a Kruskal-Wallis test) (Data Set S1, tab 2). The concordance for the top 20 most abundant pathways and resistance genes in samples collected using different methods was generally good (Fig. S3C to F). Two-sided Wilcoxon rank sum tests showed no difference between samples collected with any method frozen on day 0 and GS samples in the top 20 pathways and the top 20 resistance genes (all *P* > 0.05) (Data Set S1, tab 1).

10.1128/mSphere.00636-21.4 FIG S3 (A) Concordance evaluation of four major phyla (red-underlined box) and 15 microbial diversity metrics, including 5 at the species level (green-underlined box), 5 at the gene level (blue-underlined box), and 5 at the pathway level (gray-underlined box). The asterisks on some bars represent a *P* value of <0.05 for the corresponding measurement indices for intraclass correlation coefficients (ICCs) using a two-sided test. (B) Intermethod β-diversity dissimilarities between each method (day 0) and the gold standard (GS) based on Bray-Curtis distance at the species level. (C to F) Concordance evaluation of the top 20 most abundant pathways (C), all pathways (D), the top 20 most abundant resistance genes (E), and all resistance genes (F) using ICCs. Download FIG S3, PDF file, 1.74 MB. Copyright © 2021 Guan et al. 2021 Guan et al. https://creativecommons.org/licenses/by/4.0/ This content is distributed under the terms of the Creative Commons Attribution 4.0 International license.

### (iii) Reliability compared to the GS on day 4.

**FIG 3:**
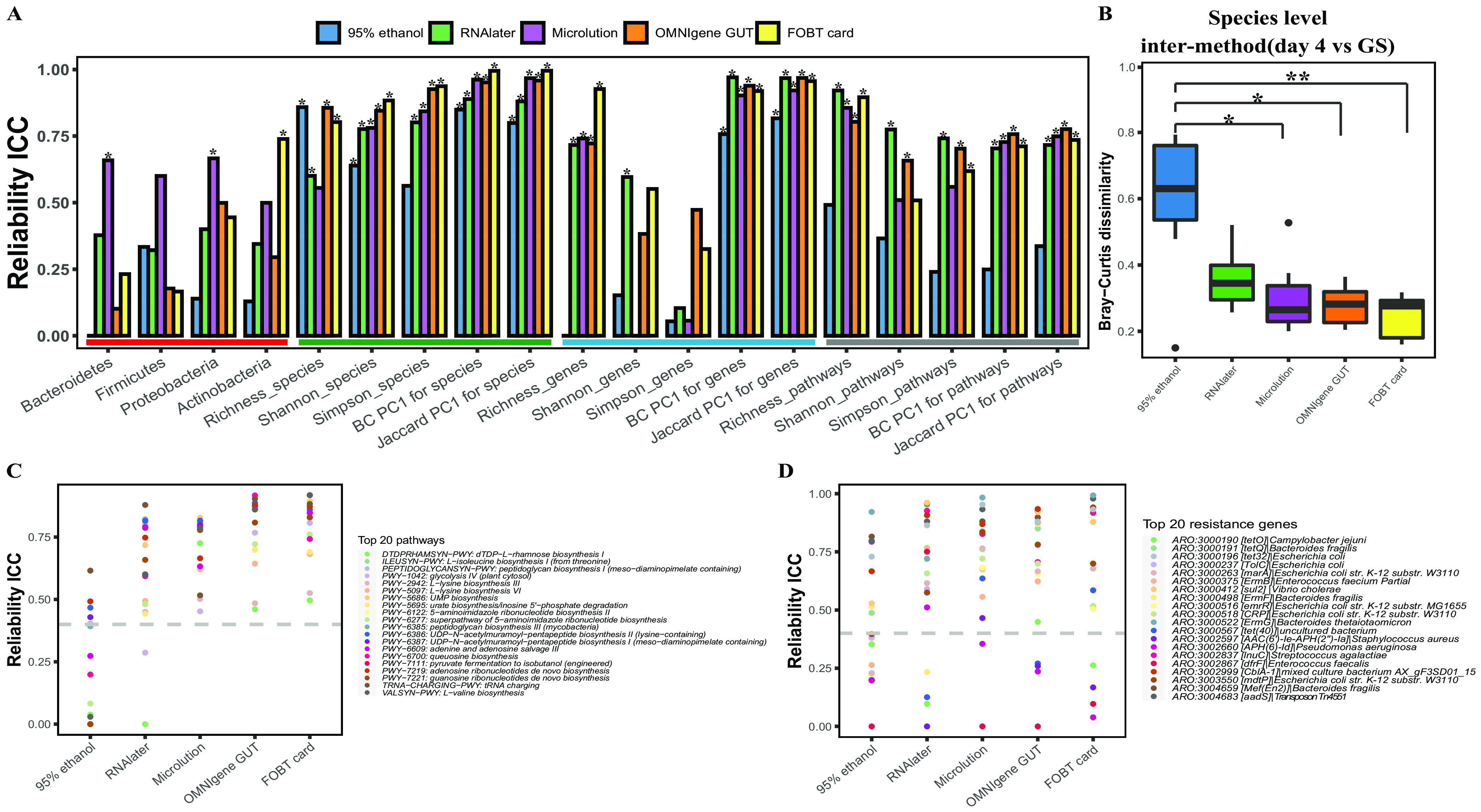
Reliability evaluation of the microbiome. (A) Microbiome metrics calculated using intraclass correlation coefficients (ICCs) included the abundances of four major phyla (red-underlined box) and 15 diversity metrics, including 5 at the species level (green-underlined box), 5 at the gene level (blue-underlined box), and 5 at the pathway level (gray-underlined box). The asterisks on some bars represent a *P* value of <0.05 for the corresponding measurement indices for ICCs using a two-sided test. (B) Intermethod β-diversity dissimilarities between each method (day 0) and the gold standard (GS) based on Bray-Curtis distance at the species level. Box plots show the intermethod Bray-Curtis dissimilarities at different time points. Each panel indicates Bray-Curtis dissimilarities between samples collected with the GS and those collected with another method. Each box represents the range between the first and the third quartiles, and the vertical lines inside the boxes represent the medians. Kruskal-Wallis tests were performed among multiple-comparison groups, and *post hoc* Dunn’s multiple-comparison tests were performed on Bray-Curtis dissimilarities between samples collected with each method and frozen on day 4 and the GS samples. * represents an FDR-adjusted Dunn *P* value of <0.05; ** represents an FDR-adjusted Dunn *P* value of <0.01. (C and D) Evaluation of the 20 most abundant pathways (C) and resistance genes (D) using ICCs.

In general, the ICC values were various, indicating high variation in reliability. The OMNIgene Gut and FOBT cards had high reliability in microbial diversity metrics (all ICCs were ≥0.75 [*P* < 0.05]) (Fig. 3A). Most methods showed low reliability in the abundances of the top 4 phyla (most ICCs were <0.4), except for Microlution (Fig. 3A). Microbial diversity indices at the species level, β-diversity (Bray-Curtis and Jaccard distance PC1) at the gene and pathway levels, and observed richness at the pathway level had high reliability (most ICCs were ≥0.75) for all collection methods. Two α-diversity indices (Shannon and Simpson) at the gene level showed low reliability (most ICCs were <0.4) for all collection methods (Fig. 3A). Two-sided Wilcoxon rank sum tests showed significant differences (*P* < 0.05) (Data Set S1, tab 1) between samples frozen on day 4 and the GS samples in the following microbial composition and diversity indices: the relative abundances of the top 4 phyla, observed richness and Shannon index at the gene level, and microbial diversity at the pathway level when collected with 95% ethanol and the relative abundances of *Bacteroidetes*, *Firmicutes*, and *Actinobacteria* and two α-diversity indices (Shannon and Simpson) at the gene level when collected with RNAlater. Samples frozen on day 4 and collected with OMNIgene Gut, FOBT cards, and Microlution had no significant difference in the preservation of the relative abundances of the top 4 phyla and microbial diversity indices at the species, gene, and pathway levels in comparison to GS samples (*P* > 0.05 by two-sided Wilcoxon rank sum tests) (Data Set S1, tab 1). The *Bacteroidetes* were underrepresented and the *Firmicutes*, *Proteobacteria*, and *Actinobacteria* were overrepresented in fecal samples collected with 95% ethanol and frozen on day 4 compared to GS samples (Data Set S1, tab 3). The Bray-Curtis dissimilarity of gut microbial species between samples collected using 95% ethanol and frozen on day 4 and the GS was greater than those between samples using other methods and the GS (Fig. 3B). There were differences among all collection methods on day 4 in the abundances of *Bacteroidetes*, *Firmicutes*, and *Actinobacteria* (FDR-adjusted *P* value of <0.05 by a Kruskal-Wallis test) (Data Set S1, tab 4). Significant differences (FDR-adjusted Dunn *P* value of <0.05) in pairwise comparisons of different methods are presented in Data Set S1, tab 5.

FOBT cards had the best performance in preserving the abundances of pathways, followed by OMNIgene Gut, RNAlater, and Microlution, while 95% ethanol had the worst performance (Fig. 3C; Fig. S4A). Significantly lower abundances were observed in 14 pathways in fecal samples collected with 95% ethanol and frozen on day 4 than in GS samples (*P* < 0.05 by two-sided Wilcoxon rank sum tests) (Data Set S1, tab 3). For the top 20 most abundant resistance genes, Microlution performed the best (almost all ICCs were >0.4), followed by OMNIgene Gut, FOBT cards, and RNAlater, while 95% ethanol performed the worst (half of the ICCs were <0.4) (Fig. 3D; Fig. S4B). Significantly lower abundances were observed for *ARO:3000191* (*tetQ*) and *ARO:3000498* (ErmF) and higher abundances were observed for *ARO:3000518* (CRP) in fecal samples collected with 95% ethanol and frozen on day 4 than in the GS samples (*P* < 0.05 by two-sided Wilcoxon rank sum tests) (Data Set S1, tab 3). As an alternative to ICCs to assess the concordance and reliability estimates, Spearman’s rank-order correlation coefficients (SCCs) were calculated, and the results were generally consistent with those of ICCs (Fig. S5).

10.1128/mSphere.00636-21.5 FIG S4 Reliability evaluation of all pathways (A) and resistance genes (B) using intraclass correlation coefficients (ICCs). Download FIG S4, PDF file, 0.8 MB. Copyright © 2021 Guan et al. 2021 Guan et al. https://creativecommons.org/licenses/by/4.0/ This content is distributed under the terms of the Creative Commons Attribution 4.0 International license.

10.1128/mSphere.00636-21.6 FIG S5 Concordance (A) and reliability (B) of four major phyla (red-underlined box) and 15 microbial diversity metrics, including 5 at the species level (green-underlined box), 5 at the gene level (blue-underlined box), and 5 at the pathway level (gray-underlined box), evaluated using Spearman correlation coefficients (SCCs). The asterisks on some bars represent a *P* value of <0.05 for the corresponding measurement indices for SCCs. Download FIG S5, PDF file, 1 MB. Copyright © 2021 Guan et al. 2021 Guan et al. https://creativecommons.org/licenses/by/4.0/ This content is distributed under the terms of the Creative Commons Attribution 4.0 International license.

### Untargeted metabolomics profiles.

After removing unnamed metabolites, a total of 1,998 metabolites were used for analysis. The number of metabolites detected in at least one fecal sample was 1,992 with 95% ethanol first, followed by 1,986 with the GS, RNAlater, or Microlution and 1,929 with OMNIgene Gut (Fig. S6A). Specifically, we classified metabolites as lipids (352; 17.6%), peptides (77; 3.9%), or amino acids (54; 2.7%); smaller percentages of detected metabolites were nucleotides (18; 0.9%), cofactors and vitamins (14; 0.7%), carbohydrates (9; 0.5%), xenobiotics (3; 0.2%), or energy (1; 0.1%), and approximately 73.6% of metabolites were unnamed at the ≥80% detectability level. The 95% ethanol method showed the best performance in amino acids, lipids, nucleotides, and peptides, while Microlution was the best in preserving carbohydrates, cofactors and vitamins, and xenobiotics (Fig. S7).

10.1128/mSphere.00636-21.7 FIG S6 (A and B) Venn diagrams of metabolites detected in at least one fecal sample (A) and with ≥80% detectability (B) captured or uncaptured by each method. (C to F) Venn diagrams of metabolites overlapping between samples collected with 95% ethanol (C), RNAlater (D), Microlution (E), and OMNIgene Gut (F) and gold-standard (GS) samples at the ≥80% detectability level. Download FIG S6, TIF file, 2.7 MB. Copyright © 2021 Guan et al. 2021 Guan et al. https://creativecommons.org/licenses/by/4.0/ This content is distributed under the terms of the Creative Commons Attribution 4.0 International license.

10.1128/mSphere.00636-21.8 FIG S7 Reliability for classified metabolites in each method estimated using intraclass correlation coefficients (ICCs) at the ≥80% detectability level. The named classified metabolites include 54 metabolites for amino acids, 9 metabolites for carbohydrates, 14 metabolites for cofactors and vitamins, 1 metabolite for energy, 352 metabolites for lipids, 18 metabolites for nucleotides, 77 metabolites for peptides, and 3 metabolites for xenobiotics. Download FIG S7, PDF file, 0.01 MB. Copyright © 2021 Guan et al. 2021 Guan et al. https://creativecommons.org/licenses/by/4.0/ This content is distributed under the terms of the Creative Commons Attribution 4.0 International license.

Four methods with preservation at multiple levels were compared by further restriction to the subsets of metabolites with ≥50% detectability (e.g., measured in ≥4 out of 8 participants), ≥80% detectability, and 100% detectability (Table 1). When metabolites were limited to the subset with ≥80% detectability, there were 1,878 metabolites in fecal samples collected with 95% ethanol first, followed by 1,867 with the GS, 1,847 with Microlution, 1,752 with RNAlater, and 1,680 with OMNIgene Gut (Fig. S6B). Compared with the GS, which had 1,867 metabolites with 80% detectability in fecal samples, 95% ethanol showed the most identical metabolites (*n* = 1,848; 99.0%). The second-best overlap of detected metabolites was observed for Microlution (*n* = 1,811; 97.0%), followed by RNAlater (*n* = 1,729; 92.6%) and OMNIgene Gut (*n* = 1,624; 87.0%) (Fig. S6C to F).

**TABLE 1:** Comparison of the numbers of metabolites at multiple detectability levels across different collection methods^*a*^

Detectability level of metabolites (%)	Method	No. of known metabolites	No. of known metabolites shared by GS and method (% of GS)	No. of known metabolites shared by GS and method on day 0	No. of known metabolites shared by GS and method on day 4
All metabolites	Immediate freezing	1,986			
	95% ethanol	1,992	1,983 (99.8)	1,977	1,980
	RNAlater	1,986	1,977 (99.5)	1,970	1,964
	Microlution	1,986	1,979 (99.6)	1,975	1,970
	OMNIgene Gut	1,929	1,921 (96.7)	1,892	1,913

≥50	Immediate freezing	1,952			
	95% ethanol	1,948	1,941 (99.4)	1,933	1,932
	RNAlater	1,883	1,879 (96.3)	1,889	1,858
	Microlution	1,913	1,905 (97.6)	1,912	1,895
	OMNIgene Gut	1,787	1,768 (90.6)	1,772	1,776

≥80	Immediate freezing	1,867			
	95% ethanol	1,878	1,848 (99.0)^*b*^	1,849	1,847
	RNAlater	1,752	1,729 (92.6)^*b*^	1,685	1,691
	Microlution	1,847	1,811 (97.0)^*b*^	1,796	1,793
	OMNIgene Gut	1,680	1,624 (87.0)^*b*^	1,617	1,612

100	Immediate freezing	1,772			
	95% ethanol	1,745	1,698 (95.8)	1,734	1,727
	RNAlater	1,230	1,206 (68.1)	1,456	1,299
	Microlution	1,659	1,602 (90.4)	1,643	1,656
	OMNIgene Gut	1,469	1,398 (78.9)	1,452	1,479

a GS, gold standard.

b The subset of known metabolites shared by the GS and other collection methods for comparison analysis of stability, concordance, and reliability at the ≥80% detectability level.

**FIG 4:**
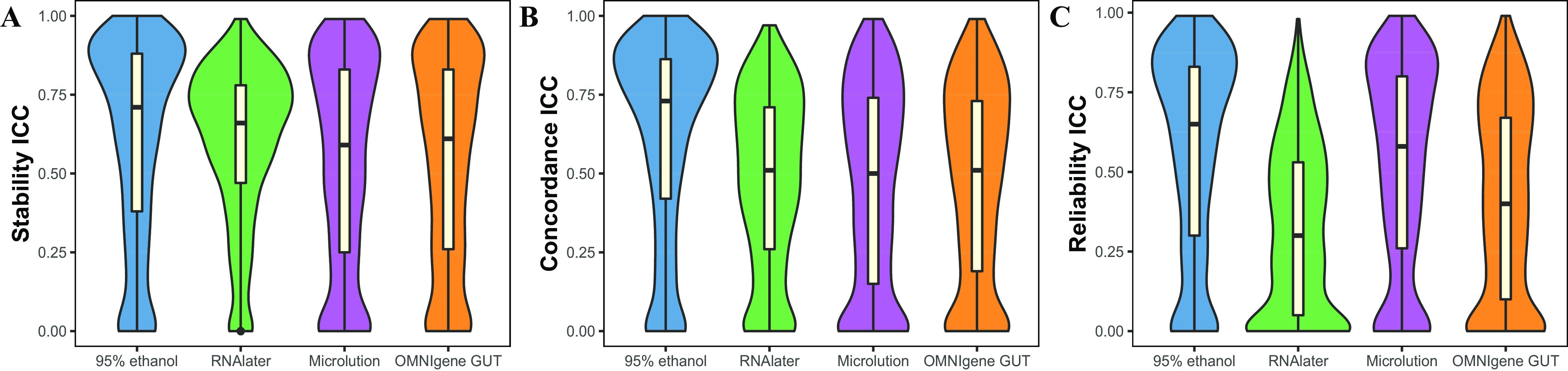
Evaluation of metabolites using intraclass correlation coefficients (ICCs) for metabolites with ≥80% detectability. (A) Stability; (B) concordance; (C) reliability. Missing values were imputed with 1/2 minimum values for a given metabolite within one method. Log_10_ transformation was used. Violin plots highlight medians and IQRs. Known (named) metabolites shared by the gold standard (GS) and the comparator method are indicated (1,848 metabolites for 95% ethanol, 1,729 metabolites for RNAlater, 1,811 metabolites for Microlution, and 1,624 metabolites for OMNIgene Gut).

At the ≥80% detectability level, fecal samples collected with 95% ethanol presented the highest ICCs across different metabolites (medians [interquartile ranges {IQRs}] of 0.71 [0.38 to 0.88] for stability, 0.73 [0.42 to 0.86] for concordance, and 0.65 [0.30 to 0.83] for reliability). For stability and concordance, the performances of the other methods were consistent (median ICCs ranged from 0.50 to 0.65). For reliability, the lowest ICCs were observed for OMNIgene Gut (0.40 [0.10 to 0.67]) and RNAlater (0.30 [0.05 to 0.53]) (Fig. 4). In addition, the medians (IQRs) of SCCs across metabolites were similar to those of ICCs in the concordance and reliability estimates, respectively (Data Set S1, tab 6). When the subset of metabolites was limited to the 100% detectability level in the stability, concordance, and reliability analyses, the results were consistent with those at the 80% detectability level (Data Set S1, tab 7).

### Functional microbes and metabolites related to SCFAs and SBAs.

**FIG 5:**
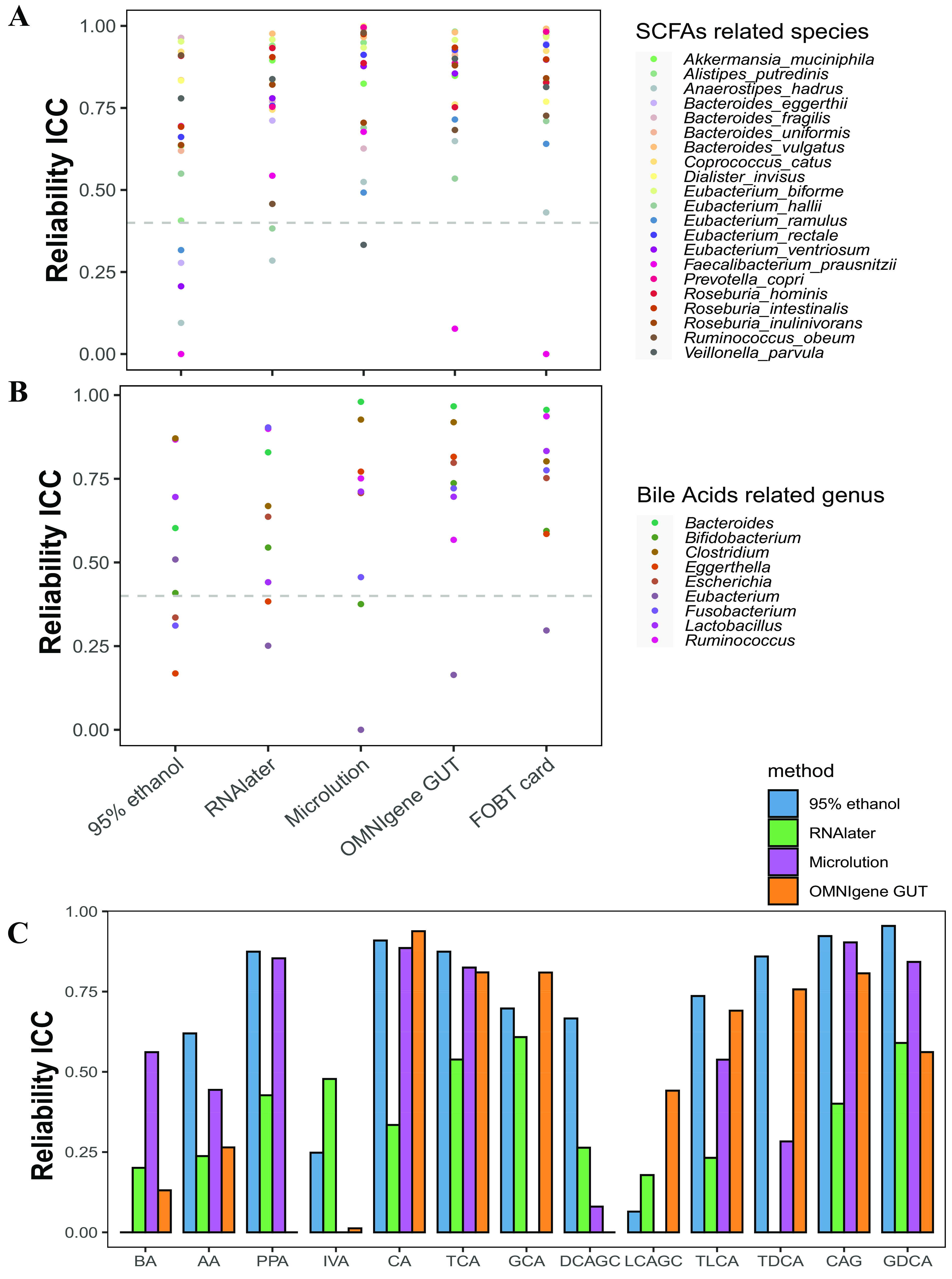
Intraclass correlation coefficients (ICCs) for reliability of the relative abundances of the functional species involved in short-chain fatty acid (SCFA) production (A), functional genera involved in secondary bile acid (SBA) metabolism (B), and their related metabolites (C). Abbreviations: BA, butyric acid; AA, acetic acid; PPA, propionic acid; IVA, isovaleric acid; CA, caproic acid; TCA, taurocholic acid; GCA, glycocholic acid; DCAGC, deoxycholic acid-glycine conjugate; LCAGC, lithocholic acid-glycine conjugate; TLCA, taurolithocholic acid; TDCA, tauroursodeoxycholic acid; CAG, cholic acid glucuronide; GDCA, glycochenodeoxycholic acid 3-glucuronide.

We searched 21 species involved in SCFA production and 9 genera involved in SBA metabolism by peer review (Table S1). Reliability scatterplots showed that FOBT cards had excellent reliability in most SCFA-related species (16 of 21 species) and SBA-related genera (6 of 9 genera), followed by OMNIgene Gut (16 species and 4 genera), Microlution (14 and 4), RNAlater (15 and 3), and 95% ethanol (8 and 2) (Fig. 5A and B). Regarding metabolites (5 SCFAs and 8 SBAs), the GS and 95% ethanol collection methods had 100% detectability for them. RNAlater and Microlution had ≥93% detectability for most metabolites, except for 44% detectability for deoxycholic acid-glycine conjugate for RNAlater. Deoxycholic acid-glycine conjugate is a bile acid-glycine conjugate, and it is produced by microbial flora. It solubilizes fats for absorption in the colon. OMNIgene Gut had poor detectability for some metabolites, such as none for propionic acid, 44% for acetic acid, and 6% for deoxycholic acid-glycine conjugate (Data Set S1, tab 8). For SCFAs, 95% ethanol showed the highest reliability ICCs (0.91 for caproic acid, 0.87 for propionic acid, and 0.62 for acetic acid), followed by Microlution (ICCs = 0.89, 0.85, and 0.44). As for SBAs, 95% ethanol had excellent reliability for half of the SBAs (4 of 8 SBAs). In addition, OMNIgene Gut showed a significant advantage in the preservation of glycocholic acid- and lithocholic acid-glycine conjugate (Fig. 5C).

10.1128/mSphere.00636-21.9 TABLE S1 Peer-reviewed literature references for species involved in SCFA production and genera involved in SBA metabolism. Download Table S1, DOCX file, 0.02 MB. Copyright © 2021 Guan et al. 2021 Guan et al. https://creativecommons.org/licenses/by/4.0/ This content is distributed under the terms of the Creative Commons Attribution 4.0 International license.

### Approximate per-sample costs and performances of different collection methods.

In summary, the cost and performance of each method in metagenomics and metabolomics are shown in Table 2. The cost of laboratory consumables or labor was not included. Estimates were calculated in U.S. dollars based on list prices as of 1 December 2020. In our study, the per-sample collection cost using OMNIgene Gut was the highest, followed by Microlution, FOBT cards, and RNAlater, and the lowest cost was 95% ethanol. OMNIgene Gut performed the best in metagenomic measurements, followed by FOBT cards, RNAlater, and Microlution. Ninety-five-percent ethanol was the best in metabolomic measurement. Microlution had a medium effect on the preservation of both microbiota and metabolites in fecal samples.

**TABLE 2:** Summary of costs and results for different collection methods

Method	Per-unit cost ($)^*a*^	Result^*d*^					
		Metagenomics			Metabolomics		
		Stability	Concordance	Reliability	Stability	Concordance	Reliability
95% ethanol	0.23^*b*^	−	++	−	++	++	+
RNAlater	5.40^*b*^	++	++	+	+	+	−
Microlution	12.14^*c*^	+	++	+	+	+	+
OMNIgene	20.00	++	++	++	+	+	−
FOBT card	7.52^*c*^	++	+	+	NA	NA	NA

a Costs for all methods were derived from Fisher Scientific, except for Microlution and OMNIgene Gut. The cost estimate for Microlution was sourced from https://www.biomart.cn/infosupply/48662146.htm. The cost estimate for OMNIgene Gut was sourced from peer-reviewed research (62).

b Two-milliliter volume; did not include containment and collection devices.

c Did not include collection device.

d ++ (good) means that the number of ICCs of ≥0.75 is ≥10 in the abundance of the top 4 phyla and 15 microbial diversity metrics at the species, gene, and pathway levels or that the median ICC across metabolites is >0.70. + (medium) means that the number of ICCs of ≥0.75 is 6 to 11 in the abundance of the top 4 phyla and 15 microbial diversity metrics at the species, gene, and pathway levels or that the median ICC across metabolites is 0.45 to 0.70. − (poor) means that the number of ICCs of ≥0.75 is <6 in the abundance of the top 4 phyla and 15 microbial diversity metrics at the species, gene, and pathway levels or that the median ICC across metabolites is <0.45. NA, not applicable.

## DISCUSSION

In this study, we compared the impacts of fecal sample collection methods and storage times on shotgun metagenomics and untargeted metabolomics profiles.

Using ICCs to estimate stability, concordance, and reliability, we observed that all of the collection methods tended to perform relatively well compared with the no-solution gold-standard sample, although there was some variability by diversity metric. Specifically, the relative abundances of the top 4 phyla and the α-diversity index at the gene level tended to have lower ICCs than did the α-diversity index at the species level or β-diversity indices.

We observed that the microbial composition of samples collected with 95% ethanol changed greatly after 4 days at ambient temperature, while FOBT cards and OMNIgene Gut were reliable in preserving microbial features. Consistent with our results, Byrd et al. found that stability ICCs using WGSS data were generally lower and more variable in samples collected using 95% ethanol (18). However, several 16S amplicon sequencing studies found that 95% ethanol resulted in little change in microbial community composition and diversity over time (14, 16). The exact mechanisms underlying these differences in the results from WGSS and 16S amplicon sequencing studies are as yet unclear. OMNIgene Gut had been proven to be a strong contender used in previous microbiome studies. When comparing this method with currently commonly used storage techniques, it demonstrated comparable efficacy in maintaining the composition of the fecal microbial community structure (17, 27–29). In accordance with our findings, Song et al. also observed that OMNIgene Gut performed the best in the preservation of fecal samples by presenting the smallest dissimilarity in bacterial composition compared with that of the GS among all studied methods (14).

RNAlater had also been tested in a number of studies, but the results were controversial. The majority of studies concluded that RNAlater was an acceptable preservative for microbial analyses of fecal samples (14, 15, 18, 30), although decreased α-diversity, DNA extraction yields (31), or purity (32) or lower stability was detected in some microbiome studies (33).

The FOBT cards, or a similar FTA card, had been shown by several studies to be good stabilizers of DNA (14, 30, 32). They can collect only small amounts of stool but offer convenient mailing. One major advantage of these cards is their ability to remain stable for remarkably long periods of time at room temperature, even for more than 10 years (34). Consistent with other studies, we found that FOBT cards tend to recover a greater diversity of bacterial taxa than other preservation methods (31).

Untargeted metabolomics was also used to assess different fecal sample collection methods in this study. Our analyses at multiple detectability levels indicated that 95% ethanol had the largest overlapping set of metabolites in comparison with the GS, and our observation was consistent with those of some previous studies (13, 20). Lim et al. found that the types of metabolites detected by OMNIgene Gut were similar to those detected by the GS and remained well correlated for up to 21 days (35). However, OMNIgene Gut had many fewer detected metabolites in comparison with the GS than samples stored in 95% ethanol and FTA cards in a previous study (20). Our results pointed out the moderate stability and low reliability of OMNIgene Gut and called researchers’ attention for further suitability exploration of the use of OMNIgene Gut to collect stool samples for metabolomics profiles. Microlution, as far as is known, has not been included in the present comparative studies of methods to collect fecal samples for microbiome or metabolite analysis. Interestingly, we noted that Microlution keeps metabolites consistent with the GS better than samples stored at ambient temperature for 4 days in RNAlater or OMNIgene Gut. It is worth noting that RNAlater was a controversial solution for metabolomics profiles in previous reports (13, 20, 36, 37). In some studies, RNAlater was incompatible with MS platforms because of its high sodium sulfate content (13, 20, 36), while in other studies, MS-based metabolomics data from samples preserved with RNAlater could be used for analyses (37). In our study, RNAlater was feasible for metabolomics measures with proper preparation of samples, such as organic solvent enrichment and filtering operations to remove a series of proteins, and the results of RNAlater were generally comparable to those with other methods.

In the current study, we specifically evaluated the reliability of microbial species involved in SCFA production, genera involved in SBA metabolism, and their related metabolites. Our data suggested that different storage conditions (interaction of collection method and storage time at ambient temperature) could cause diversified fluctuations in the relative abundances of functional bacteria in a time/method-dependent or -independent way.

This study has several strengths. This was the first study, to our knowledge, that evaluated the stability and concordance of human fecal sample collection methods using WGSS and metabolomics. As the cost of sequencing decreases, there is a shift toward WGSS for microbiome studies due to the increased resolution of taxonomic and additional functional information. In addition, we specifically estimated the effect of the fecal collection method on the microbiota and metabolites related to SCFAs and SBAs. Moreover, our study was the first to assess Microlution’s effectiveness relative to other approaches, which is a recently commercially available approach for the temporary preservation of samples at ambient temperature.

Limitations of this study included the small sample size and potentially limited generalizability to other populations, as our participants were young and healthy. For example, FOBT cards may be a useful tool in large-scale stool microbiota studies in children but should not be used when studying meconium, where the stool container (Eppendorf tube) introduced more variation than the biological variation (38). Meconium samples clustered more by container than by individual and storage condition. Thus, we regarded fecal samples frozen immediately without any solution as the GS. However, this GS underwent at least one freeze-thaw cycle when frozen samples were taken out and thawed for DNA extraction, which potentially influenced the microbiome (14).

Epidemiological field sample collection and storage are also affected by different dimensions of factors, including the stool sample collection site, the influences of the storage temperature of stool samples (4°C, −20°C, or −80°C, etc.) and freeze-thaw cycles of stool samples (39), and the influence of the transportation process for the stool sample after collection (40). These dimensions will affect the changes in microbes and their metabolites in feces and cause deviations in reflecting human-microbe relationships. Subsequent quality control (QC) studies should consider evaluating more dimensional factors, as mentioned above. Future studies should also be extended to other populations (e.g., patients, infants, and the elderly) to improve generalizability and collect multiple fecal samples over a period to assess long-term stability (i.e., samples placed at −80°C for 3 months, 6 months, and 1 year, etc.). Besides, further research is needed to ascertain whether these collection methods are suitable for other omics technologies such as metatranscriptomics and metaproteomics.

### Conclusion.

Our study suggests that when preserving fecal samples at ambient temperature for up to 4 days, OMNIgene Gut, FOBT cards, RNAlater, and Microlution were good collection methods in gut microbiome studies, and 95% ethanol is recommended for fecal metabolome studies. We recommend using separate collection methods for gut metagenomic sequencing and fecal metabolomic profiling in large population studies where preservation at ambient temperature for a few days is inevitable.

## MATERIALS AND METHODS

### Fecal specimen collection and storage.

Eight self-reported healthy subjects who were 26.0 ± 1.5 years old (mean ± SD) were recruited from Fudan University, Shanghai, China. None of the subjects had used antibiotics or probiotics within the previous 2 weeks or had ever received chemotherapy treatment. All participants provided informed consent, and the study was approved by the Institutional Review Board (IRB) at Fudan University (IRB approval no. FE20064).

A plastic bedpan was provided to each participant for self-collecting fecal samples. For each participant, the fecal specimen was manually mixed using a spatula. Single fecal specimens from each participant were divided into 11 samples. Sample 1 was placed in a feces tube (Biorise, China) without any solution as the GS. Samples 2 and 3 were separately placed into a 2-ml Eppendorf tube filled with 500 μl of 95% ethanol (Sinopharm, China) and 200 mg of zirconia/silica beads (BioSpec, USA), while samples 4 and 5 were separately placed into a tube with 500 μl of RNAlater stabilization solution (Qiagen, Germany) and 200 mg of beads. Samples 6 and 7 were separately placed into Microlution tubes (DaYunGene, China) (Microlution is a relatively new stool collection kit designed for the collection and preservation of the intestinal flora of fecal samples [41]). The description and protocol for Microlution can be found in Text S1 in the supplemental material. Samples 8 and 9 were separately placed into OMNIgene Gut tubes (DNA Genotek, Canada) according to the manufacturer’s protocol, without modifications. Once samples were collected, subjects were instructed to shake all tubes (except for the GS) for homogenization. Two double-slide Hemoccult Sensa for FOBT cards (Beckman Coulter, USA) were smeared thinly with samples 10 and 11, and the flaps were closed. In total, 88 fecal samples were self-collected for measurements and then handed over to the study administrators. Next, samples 1, 2, 4, 6, 8, and 10 from each participant were stored at −80°C within 30 min after collection (day 0), and the other samples, 3, 5, 7, 9, and 11, were placed at ambient temperature for 4 days (day 4) to simulate the effects of carrier transport (“mock shipping”). The sample collection and experimental schemes are illustrated in Fig. 1.

10.1128/mSphere.00636-21.1 TEXT S1 Description and sampling instructions of Microlution. Download Text S1, PDF file, 0.6 MB. Copyright © 2021 Guan et al. 2021 Guan et al. https://creativecommons.org/licenses/by/4.0/ This content is distributed under the terms of the Creative Commons Attribution 4.0 International license.

### DNA extraction, library preparation, and whole-genome shotgun sequencing.

Samples were thawed on ice and then vortexed completely for homogenization. For samples 1, 2, 3, 4, and 5 from each participant, approximately 150 to 200 mg of feces was retrieved using a sterile spatula and placed into a 2-ml Eppendorf tube. Two hundred microliters of the feces-solution mixture was transferred from samples 6, 7, 8, and 9 into 2-ml Eppendorf tubes. One window (a squared area containing the majority of the stool sample) from FOBT cards was cut from samples 10 and 11 and put into 2-ml Eppendorf tubes. Total DNA was extracted using the TIANamp stool DNA kit (Tiangen, China) according to the manufacturer’s instructions, with some modifications. Samples were placed into 2-ml Eppendorf tubes with lysis solution, proteinase K, and 0.25 g beating beads and heated at 75°C for 15 min, prior to bead beating for 1 min using a vortex mixer (Labnet America). The quantity of the DNA solution was assessed using an Equalbit double-stranded DNA (dsDNA) high-sensitivity (HS) assay kit (Vazyme, China) before library construction and sequencing. The Illumina sequencing libraries were constructed from 1 ng of input DNA using the Tn*5* DNA library prep kit for Illumina (APExBIO, USA) according to the manufacturer’s recommended protocol. After final library quantification control using a Qubit Flex fluorometer (Vazyme, China) and quality control (QC) using the Agilent Bioanalyzer 2200 system (Agilent Technologies, USA) to confirm the expected insert size distributions, all libraries were paired-end (150-bp reads) sequenced on the Illumina Novaseq6000 platform (Illumina, USA). Raw sequences were first quality filtered by FastQC (version 0.11.8). Kneaddata (version 0.7.2) was used to run preprocessing tools. We scanned the reads with a 4-base-wide sliding window, cutting when the average quality per base dropped below 20, and dropped reads that were <75 bases long by Trimmomatic (version 0.33). Next, all reads that mapped to the human genome GRCh37 were removed by Bowtie2 (version 2.3.4.3).

After quality control processing, the average coverage was about 32,852,947 reads per sample. The taxonomic profiles of metagenomics were determined by MetaPhlan3 (version 3.0.3). The MetaCyc Pathway profiles were determined by HUMAnN3 (version 3.0.0.alpha.3). UniRef90 and CARD (version 3.0.8) were used to create a custom antibiotic resistance gene database by ShortBRED-identify in ShortBRED (version 0.9.5), and antibiotic resistance gene profiles were determined by shortbred_quantify.py. The functional taxonomies involved in SCFA production and SBA metabolism were determined in peer-reviewed literature (42–47).

### Untargeted metabolomics profiles.

Fecal samples collected with no solution, 95% ethanol, RNAlater, OMNIgene Gut, and Microlution were freeze-dried overnight. Next, 20-mg lyophilized fecal samples were weighed, dissolved in 600 μl methanol (80%), and homogenized for 90 s with a tissue grinder (Shanghai Jing Xin, Shanghai, China) at 20 Hz. After centrifugation (18,000 × *g* for 15 min at 4°C), the supernatants were transferred, dried overnight under nitrogen flow, and stored at −80°C before preparation for analysis. Samples after nitrogen flow were resuspended with 300 μl 80% methanol (containing 5 μg/ml 2-chloro-l-phenylalanine as the internal standard). QC samples were prepared for each test separately by combining aliquots from all resuspended contents. The resuspended contents and QCs were filtered through an organic filtering membrane (diameter, 0.22 μm). The samples collected using FOBT cards were limited in amounts and therefore were not available for metabolomics analysis. The QCs were injected regularly per set of 9 samples in each analytical batch to monitor the stability and correct for instrumental drift (48). The acquisition of metabolites was performed on an Agilent 1290 Infinity II ultrahigh-performance liquid chromatography (UHPLC) system coupled to an Agilent 6545 ultrahigh-definition and accurate-mass quadrupole time of flight (Q-TOF)/MS system used for liquid chromatography-mass spectrometry (LC-MS) analysis. The column (10-cm by 2.1-mm, 2.5-μm XSelect high-strength silica T3 column) was eluted with a linear gradient of 5% solvent B over 0 to 2 min, 5 to 95% B over 2 to 10 min, and 95% B over 10 to 15 min; the post time (equilibration time of the mobile phase between two runs) was held at 5% solvent B for 5 min for system balance, where solvent A is water with 0.1% formic acid and solvent B is acetonitrile with 0.1% formic acid. The flow rate was 0.4 ml/min. All the samples were kept at 4°C during the analysis.

Mass spectrometry was operated in both the positive electrospray ionization (ESI^+^) mode and the negative ESI (ESI^−^) mode. The optimized parameters were as follows: 3.5-kV capillary voltage and 10-liter/min drying gas flow, operating in both the positive mode (ESI^+^) and the negative mode (fragmentor voltage, 45-V skimmer voltage, and mass range of *m/z* 50 to 3,000).

Raw data were converted to the mzData format by Agilent Masshunter Qualitative Analysis B.08.00 software (Agilent Technologies, USA). In the R software platform, the XCMS program was used for peak identification, retention time correction, and automatic integration pretreatment. Next, the data were subjected to internal standard normalization. Visualization matrices containing the sample name, *m/z*-retention time pair, and peak area were obtained. Eventually, the features were identified by matching the accurate *m/z* value obtained from the metabolomics analysis to the HMDB database.

### Distance metrics and contribution of variables to overall microbiota and metabolite variability.

Distance metrics were used to summarize the overall microbiota and metabolite variability. Bray-Curtis distance was generated from microbial species composition for principal-coordinate analysis (PCoA), and Euclidean distance was generated from metabolite profiles for principal-component analysis (PCA). The percentage of variability (*R*^2^) explained by subject, collection method, and storage time was estimated using the Adonis function (49, 50) in the vegan package in R to perform permutational multivariate analysis of variance.

### Statistical analysis.

We used intraclass correlation coefficients (ICCs) to quantify the stability, concordance, and reliability of different storage methods.

“Stability” was defined as a standard ICC, where $\sigma_{b}{}^{2}$ represents the between-individual variability and $\sigma_{t}{}^{2}$ represents the within-individual variability over time within each collection method (intramethod variability):

$$\frac{{\text{σ}}_{b}{}^{2}}{{\text{σ}}_{b}{}^{2}\;+{\text{σ}}_{t}{}^{2}}$$

We calculated stability by comparing samples on day 4 to the ones frozen directly after collection using the same collection method.

“Concordance” was similarly defined as an ICC where $\sigma_{b}{}^{2}$ represents the between-individual variability and $\sigma_{m}{}^{2}$ represents the variability introduced by the collection method:

$$\frac{{\text{σ}}_{b}{}^{2}}{{\text{σ}}_{b}{}^{2}\;+{\text{σ}}_{m}{}^{2}}$$

We calculated concordance by comparing samples collected by each of the five methods frozen on day 0 with GS samples.

“Reliability,” the most instructive index for selecting optimal collection methods in epidemiological fieldwork, was also defined as an ICC where $\sigma_{b}{}^{2}$ represents the between-individual variability and $\sigma_{e}{}^{2}$ captures the variability due to different collection methods and temporal instability:

$$\frac{{\text{σ}}_{b}{}^{2}}{{\text{σ}}_{b}{}^{2}\;+{\text{σ}}_{e}{}^{2}}$$

We calculated reliability by comparing fecal samples frozen after 4 days at ambient temperature using each of the five methods with GS samples. The ICCs and their corresponding *P* values were calculated using the R statistical package “irr” based on the two-way mixed-effects model, single-measures type, and consistency definition (51). ICC values of <0.4 were interpreted as representing a poor outcome, ICCs of 0.4 to 0.75 were interpreted as representing a fair to good outcome, and ICCs of ≥0.75 were interpreted as representing an excellent outcome (52).

For the microbiome, taxa, genes, and pathways present in <5% of samples (i.e., fewer than four samples here) were excluded to filter out potentially spurious features due to sequencing or classification error (53). ICCs were calculated based on the square root of the relative abundances of the top 4 phyla (i.e., *Bacteroidetes*, *Firmicutes*, *Proteobacteria*, and *Actinobacteria*) and 15 microbial diversity metrics (observed richness, Shannon index, and Simpson index for α-diversity and the top PCoA component [PC1] for Bray-Curtis distance and Jaccard distance for β-diversity), including 5 at the species level, 5 at the gene level, and 5 at the pathway level. The ICCs of the top 20 most abundant as well as all pathways and resistance genes were also calculated.

For untargeted metabolomics, after combining the ESI^+^ and ESI^−^ modes, some known metabolites will have multiple adductions. The value of the ion with the lowest relative standard deviation (RSD) for the QC samples was preferred as the value of the metabolite. The RSD was calculated using the ratio of the standard deviation over the arithmetic mean value across the QC samples. Metabolites with missing values of >50% in the QC samples were removed before analysis. Log_10_ transformation was performed before analyses. Missing values were imputed with 1/2 minimum values for a given metabolite among comparative samples. For analyses including samples collected with 95% ethanol, RNAlater, Microlution, OMNIgene Gut, or FOBT cards, metabolites were further restricted to those with ≥80% detectability in the collection method of interest. Limiting these analyses to those metabolites with high detectability ensured an adequate number of samples for calculating ICCs in a small sample size.

In our study, the reliabilities of microbial species involved in SCFA production, genera involved SBA metabolism, and their related metabolites were also calculated by ICCs.

To further determine which microbial diversity metrics differed significantly among samples collected by different methods at each time point (day 0 and day 4), the Kruskal-Wallis test (54) was performed. The Benjamini-Hochberg (BH) FDR method was applied to address multiple-comparison issues. An adjusted Kruskal-Wallis *P* value of <0.05 was considered statistically significant. Pairwise comparisons of the collection methods (e.g., 95% ethanol versus RNAlater) were conducted using *post hoc* Dunn’s multiple-comparison test (55). An FDR-adjusted Dunn *P* value of <0.05 was considered statistically significant for comparison within each pair of methods at one time point (day 0 or day 4).

As recommended for the assessment of the reliability of measurement scales (56), the ICC was calculated as our main measurement. However, when the measurements of ICCs were from different methods, they might have significantly different biases (30). Thus, when comparing microbiota and metabolites in each method with those of the GS (i.e., concordance and reliability), we further calculated an interclass correlation coefficient, i.e., Spearman’s rank-order correlation coefficient (SCC), as a secondary analysis to assess the robustness of our results to distribution assumptions.

The R packages ade4 (6), vegan (57), DESeq2 (58), phyloseq (59), irr (60), ropls (61), stats (54), and PMCMR (55) were used for the statistical analyses. All statistical analyses were performed in R version 4.1.1.
