# Supplementary figures and images for: Comparison of Fecal Collection Methods on Variation in Gut Metagenomics and Untargeted Metabolomics
Source: mSphere. 2021 Sep 15;6(5):e00636-21. doi: 10.1128/mSphere.00636-21 (PMC8550109; doi:10.1128/mSphere.00636-21)

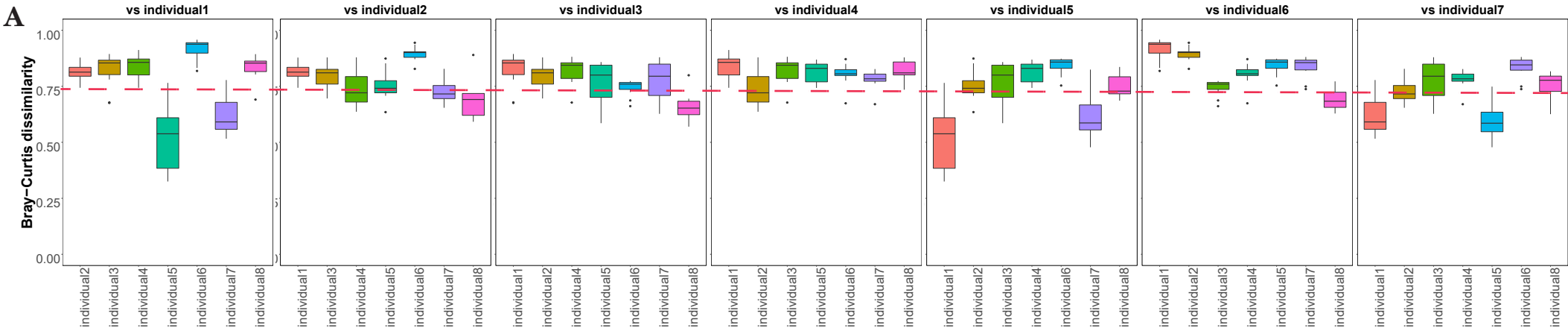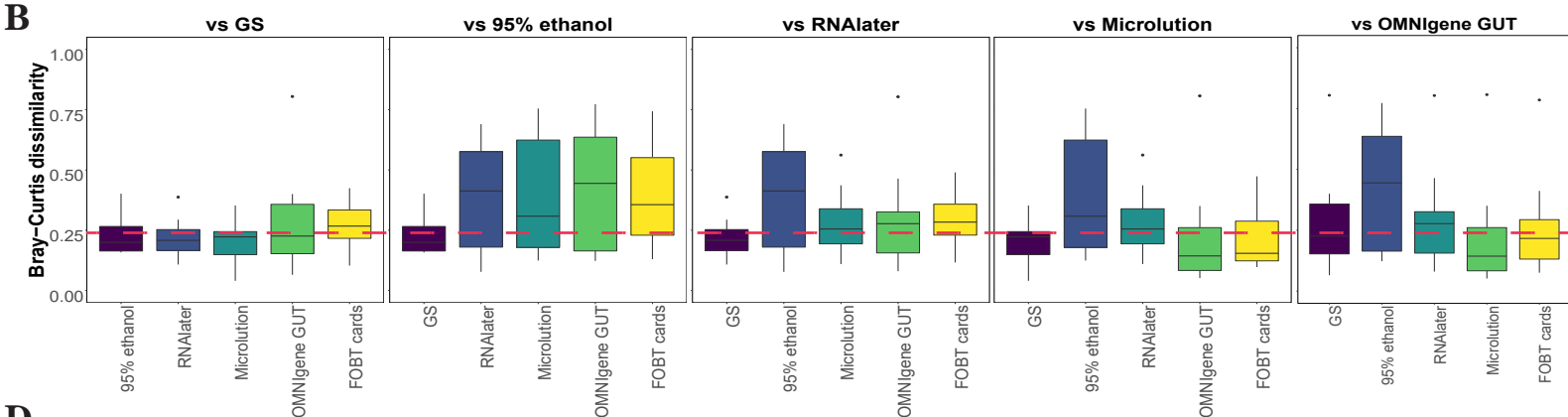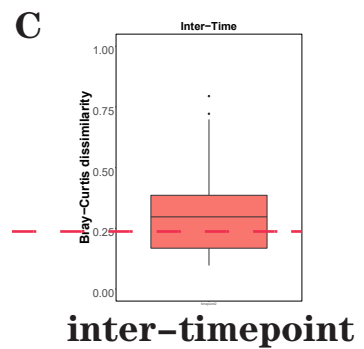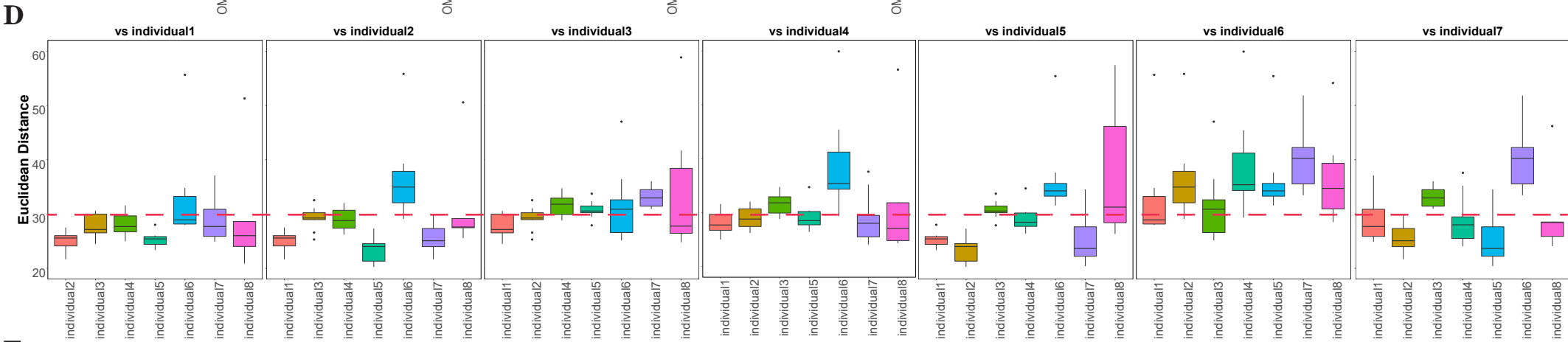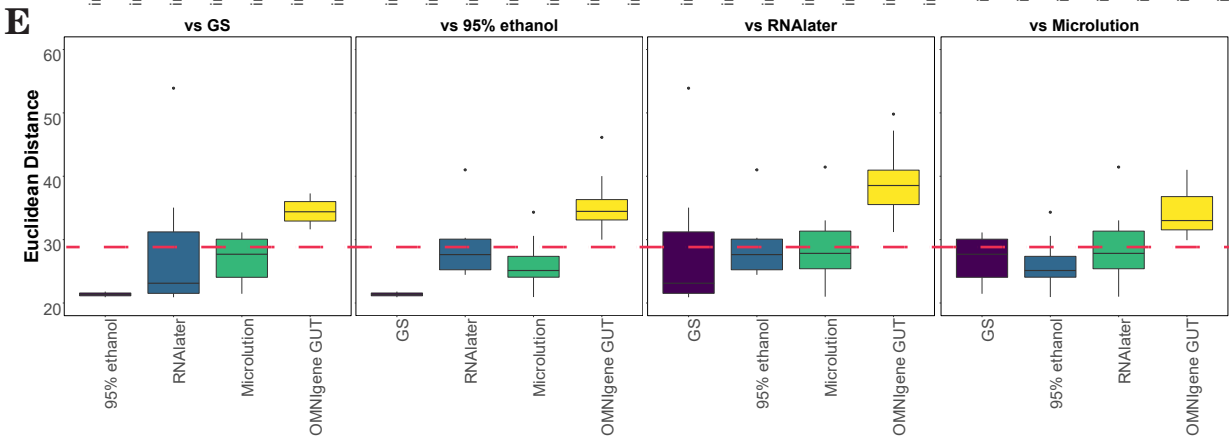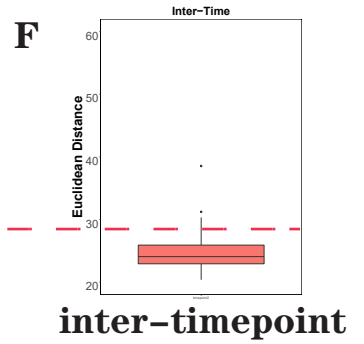

Supplement: FIG S1 [file msphere.00636-21-sf001.pdf]

A

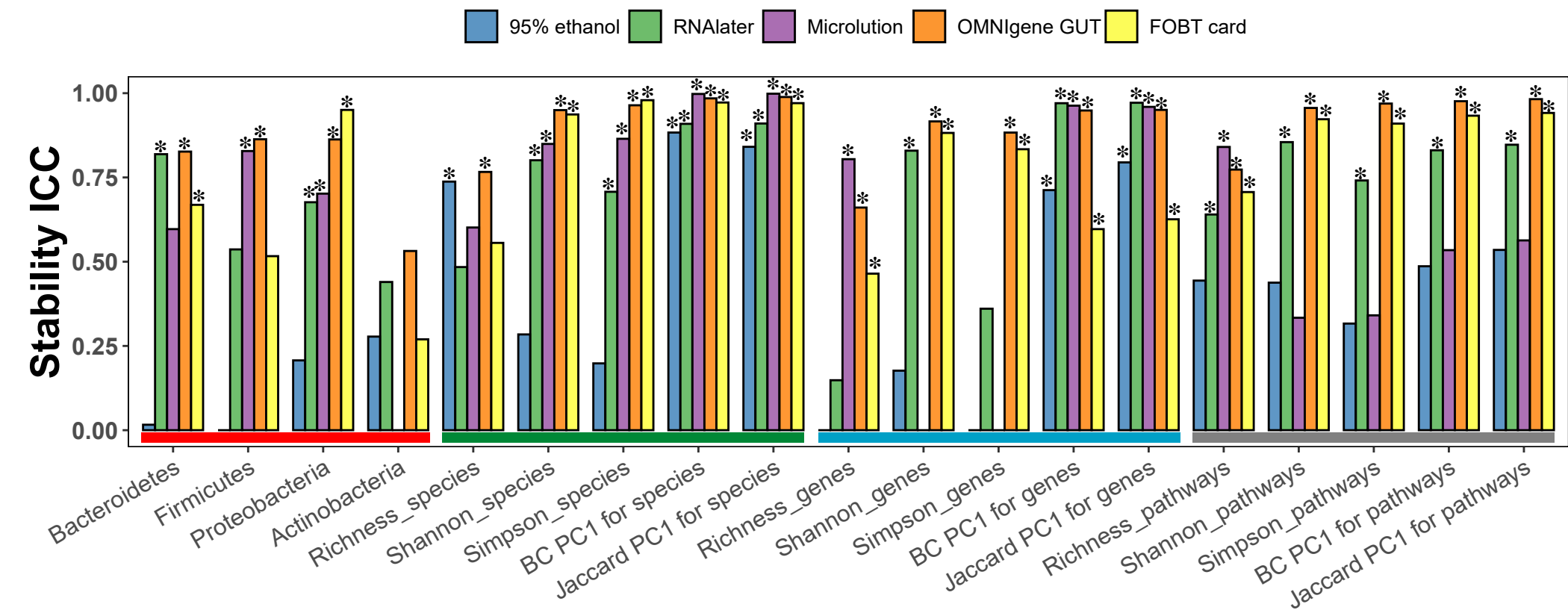

B

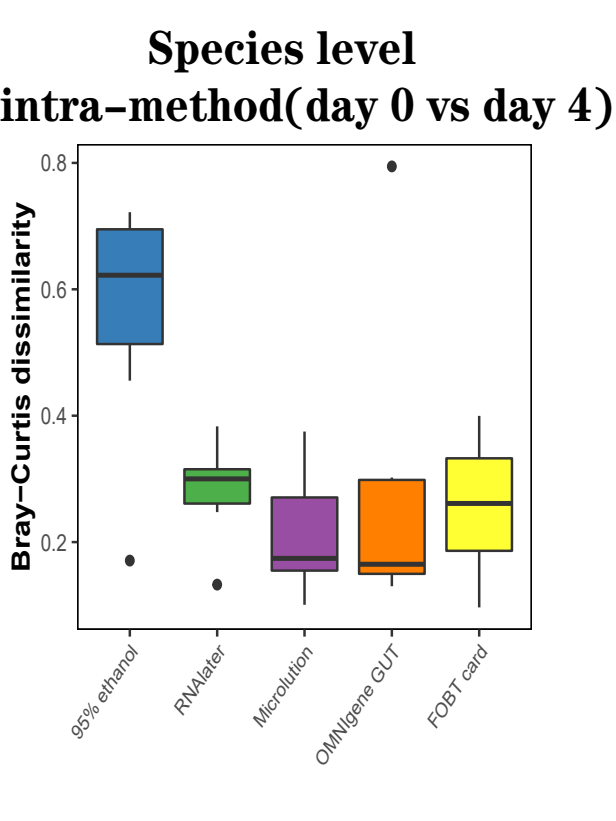

C

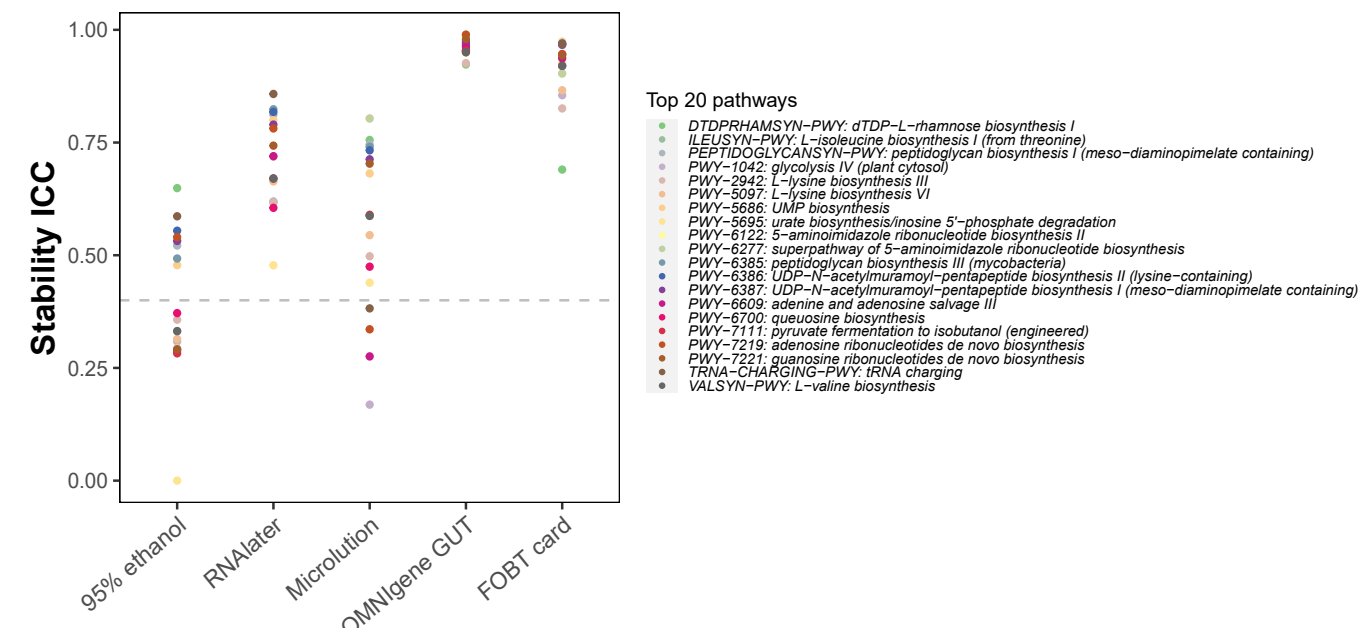

D

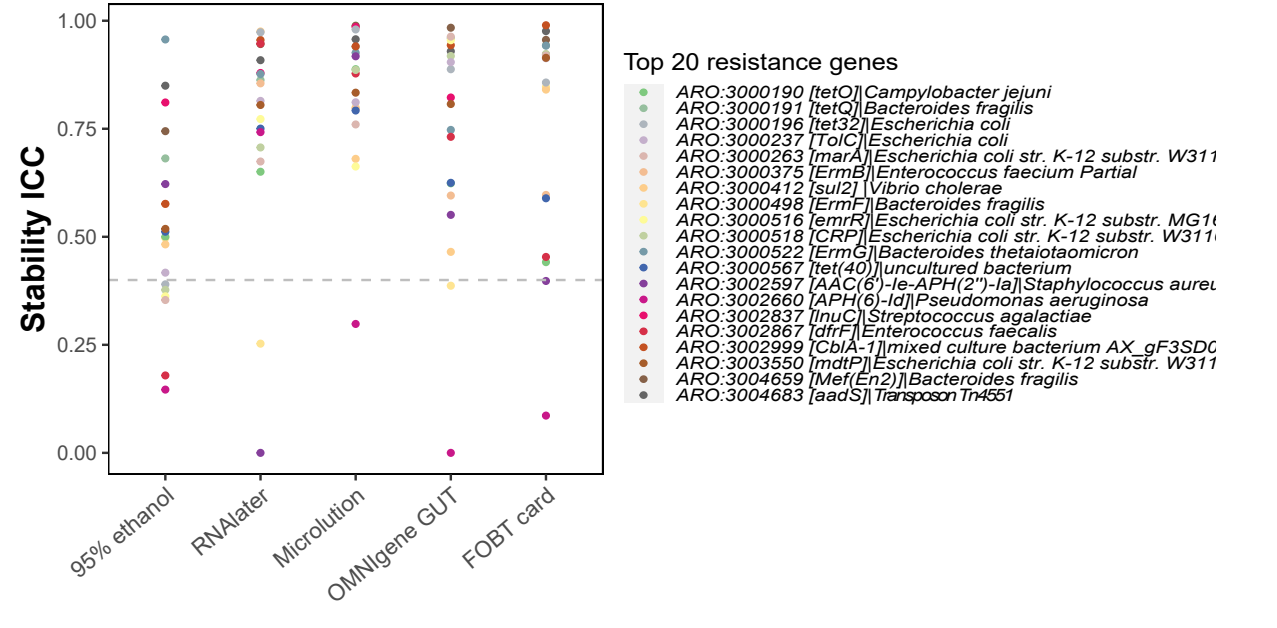

E

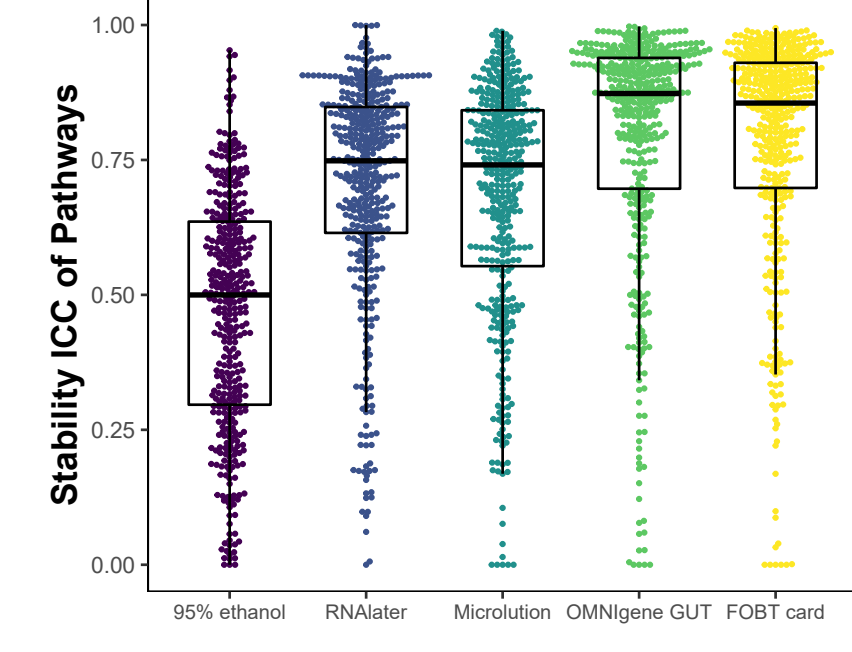

F

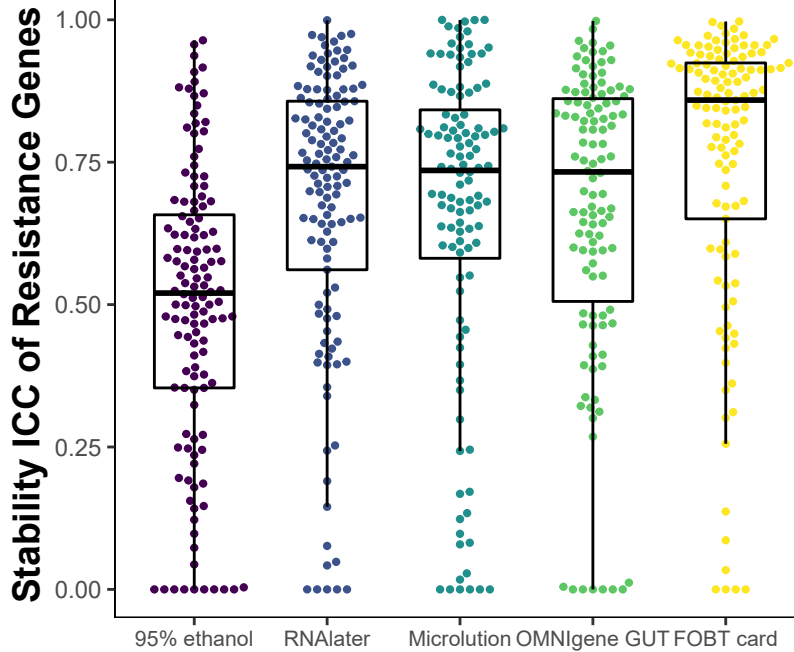

Supplement: FIG S2 [file msphere.00636-21-sf002.pdf]

A

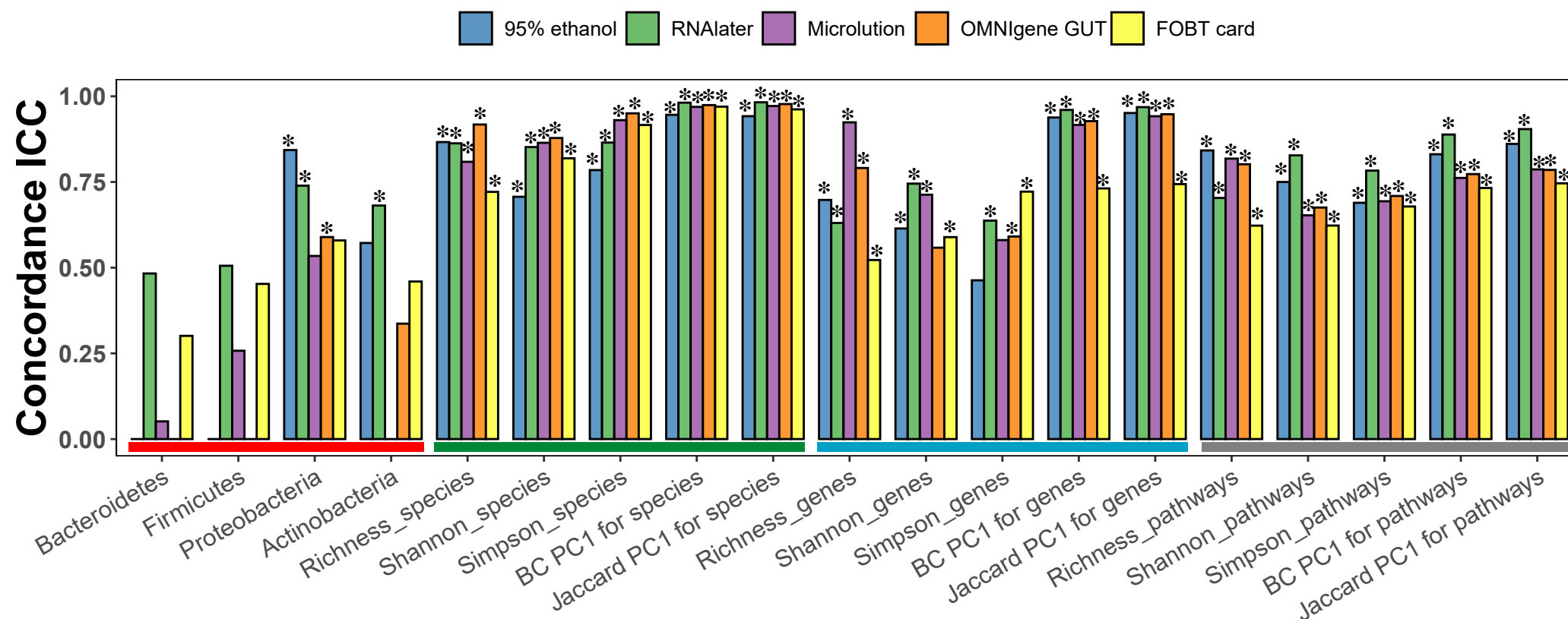

B

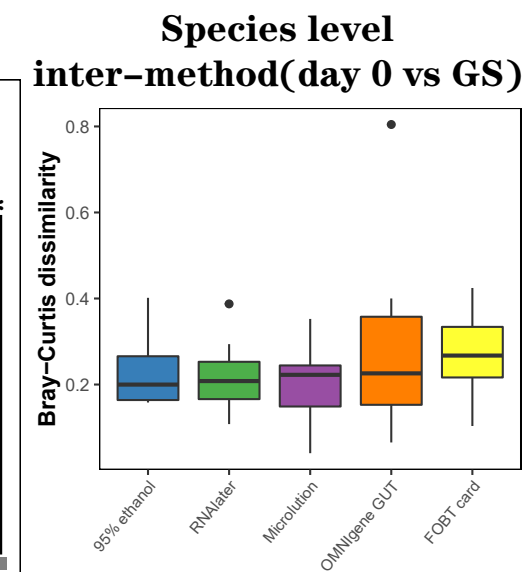

C

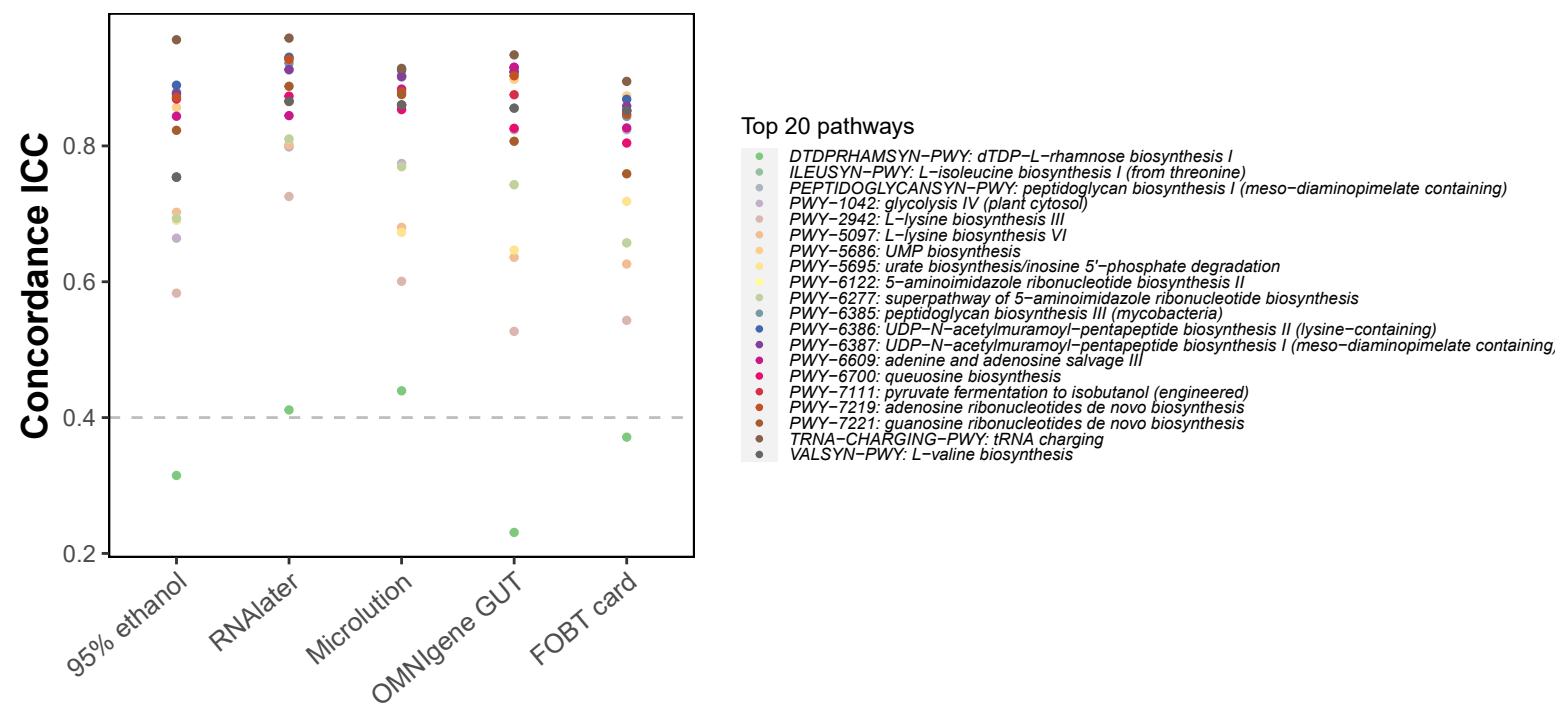

D

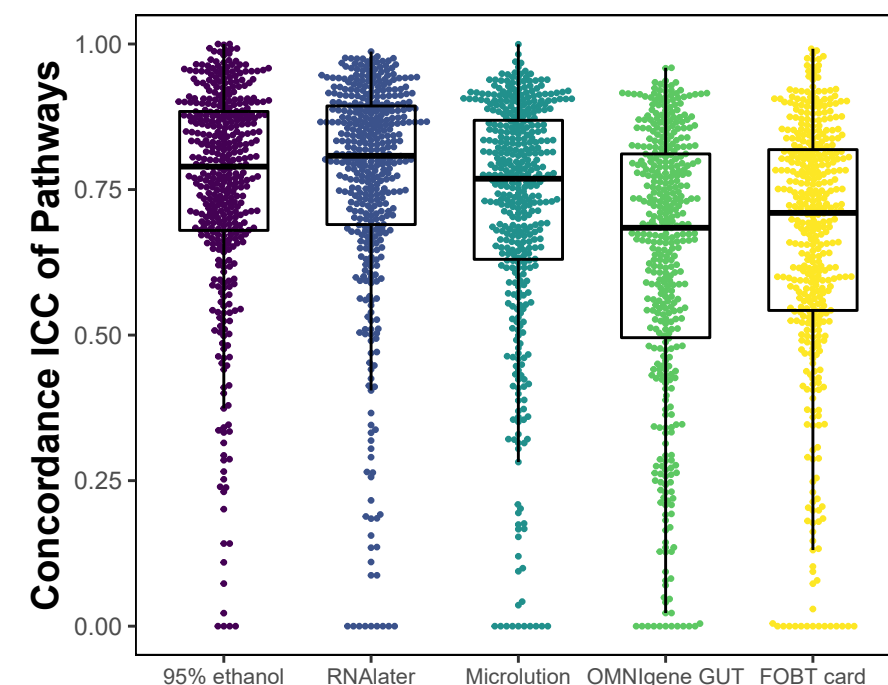

E

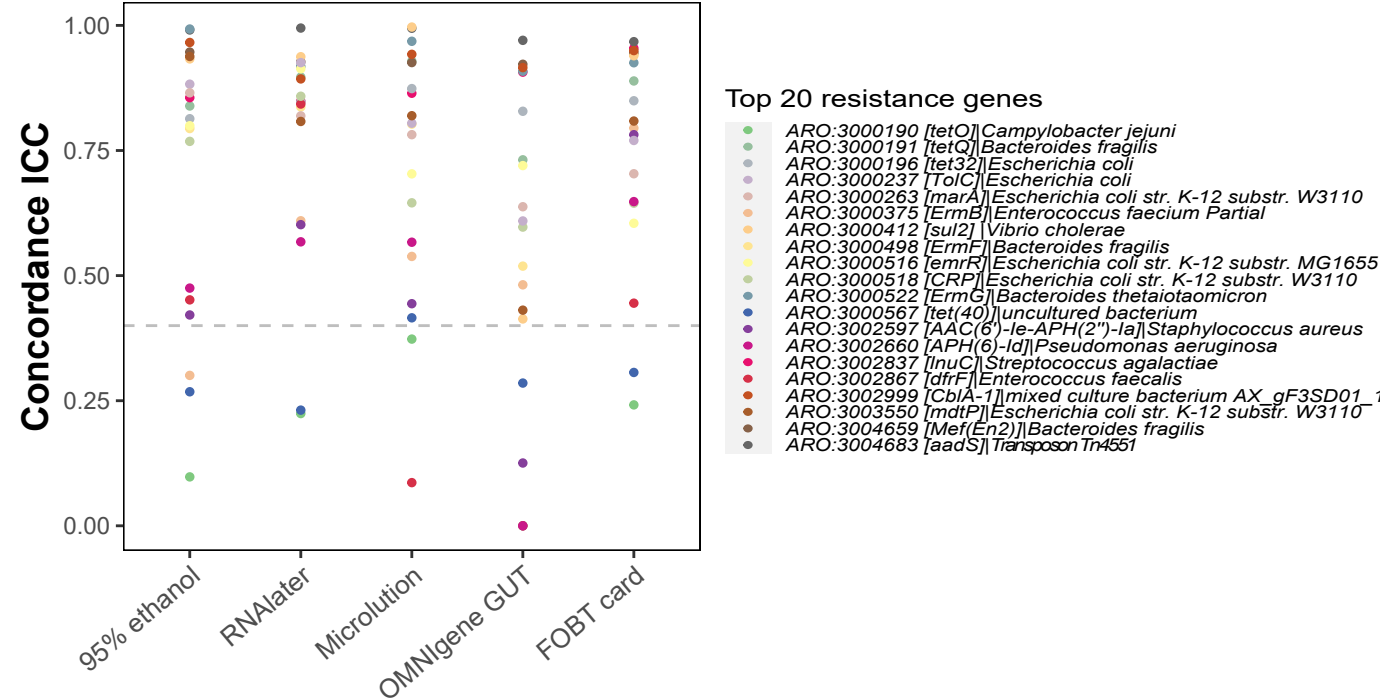

F

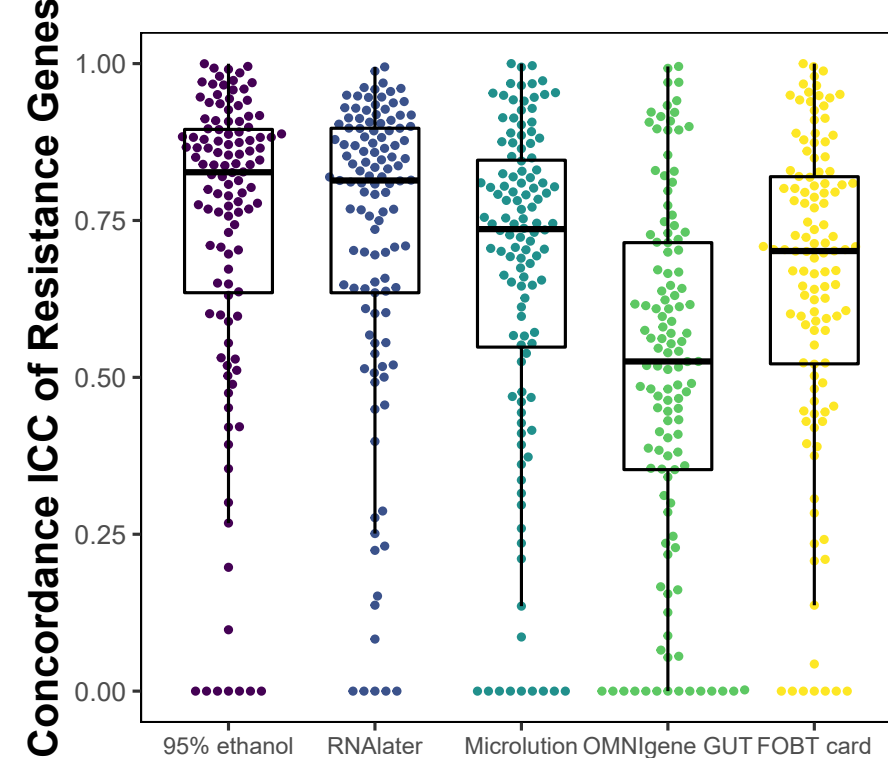

Supplement: FIG S3 [file msphere.00636-21-sf003.pdf]

**A****Reliability ICC of Pathways**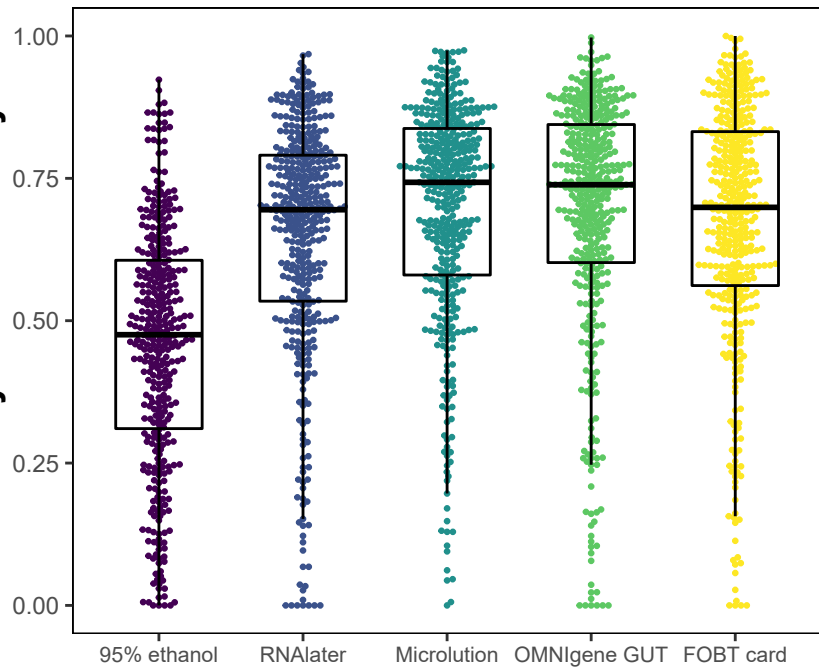**B****Reliability ICC of Resistance Genes**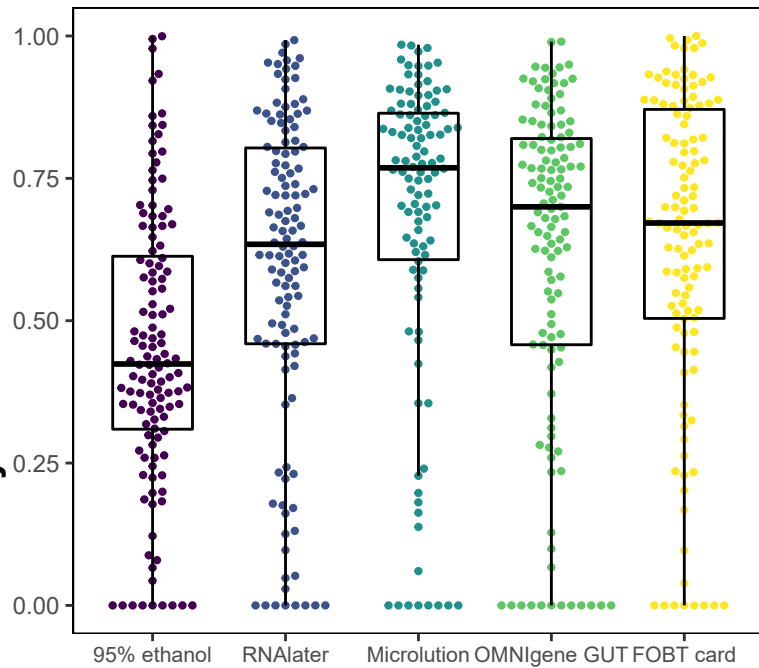

Supplement: FIG S4 [file msphere.00636-21-sf004.pdf]

**A**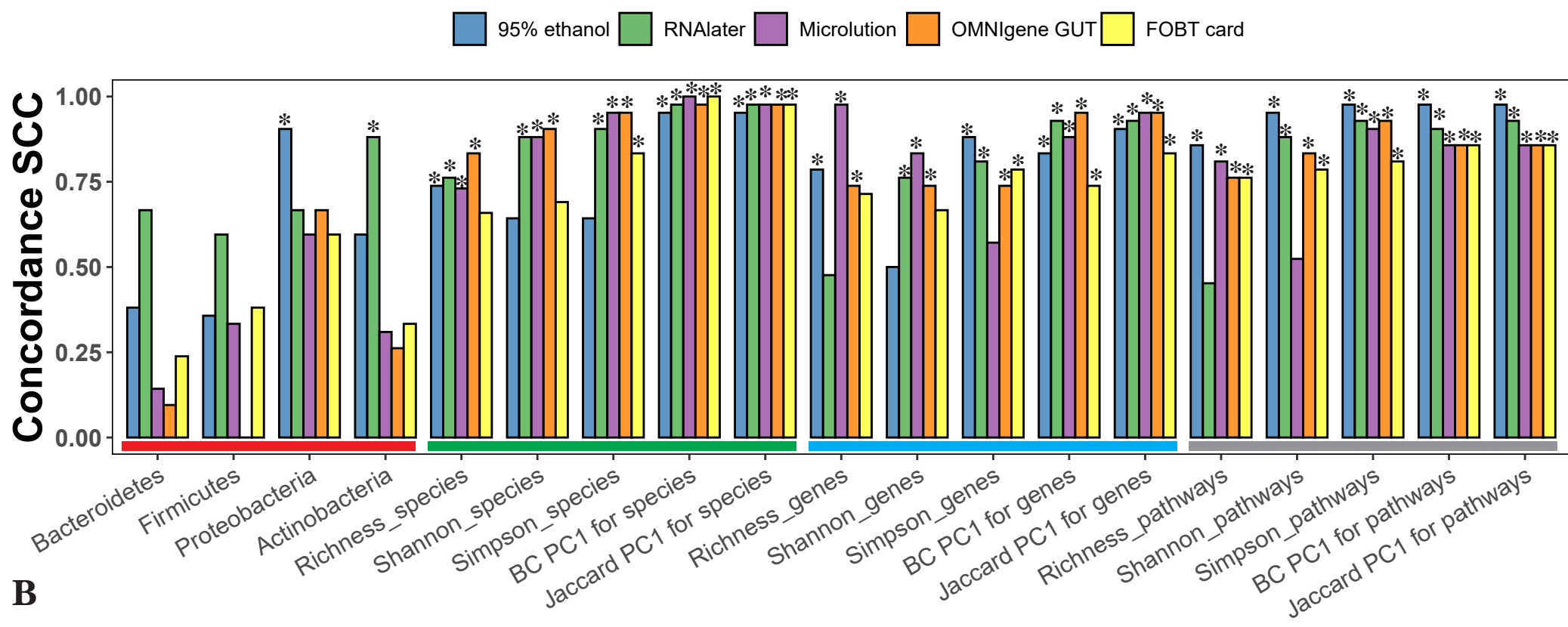**B**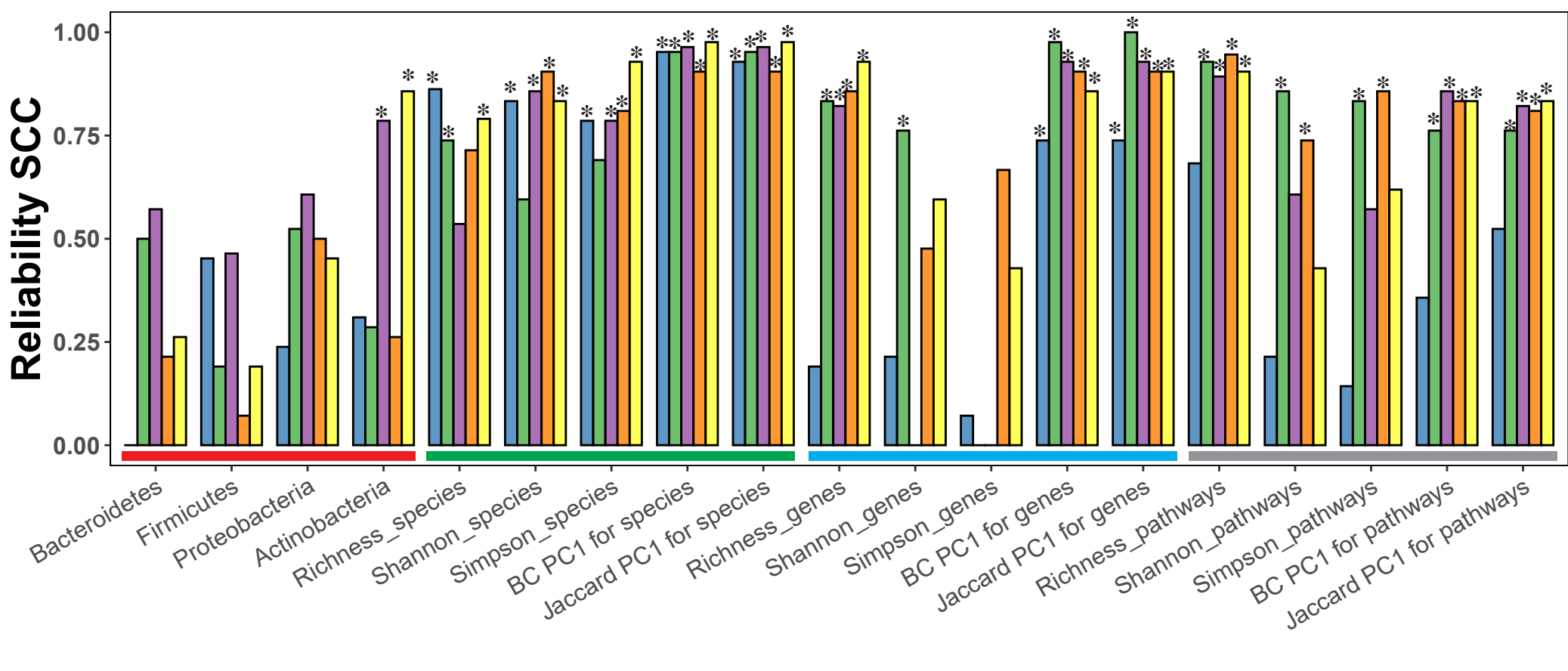

Supplement: FIG S5 [file msphere.00636-21-sf005.pdf]

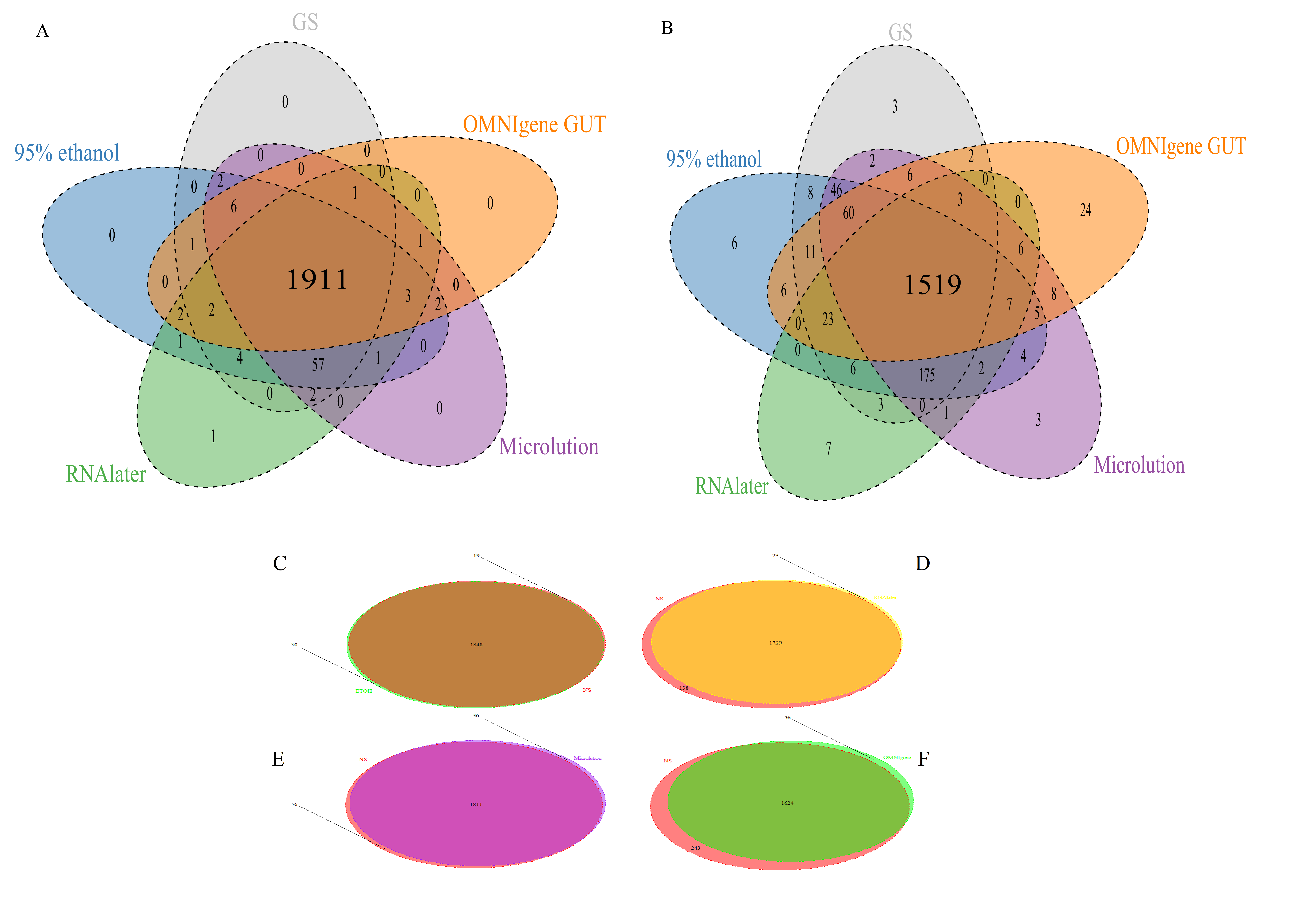

Supplement: FIG S6 [file msphere.00636-21-sf006.tif]

Reliability ICC

95% ethanol RNAlater Microtution OMNIgene GUT

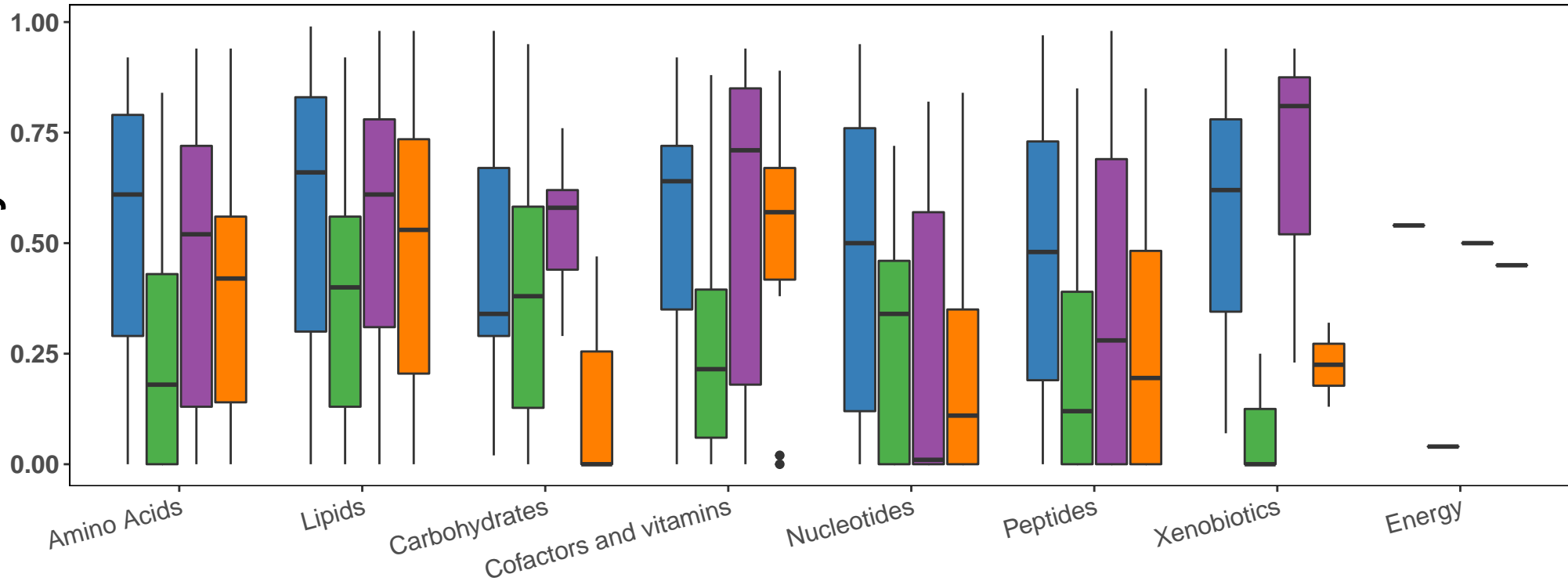

Supplement: FIG S7 [file msphere.00636-21-sf007.pdf]
