# Supplementary material for: Comparison of Fecal Collection Methods on Variation in Gut Metagenomics and Untargeted Metabolomics
Source: mSphere. 2021 Sep 15;6(5):e00636-21. doi: 10.1128/mSphere.00636-21 (PMC8550109; doi:10.1128/mSphere.00636-21)
Supplement: TABLE S1 [file msphere.00636-21-st001.docx]

**Table S1. Peer-reviewed literatures references for 21 species involved in Short Chain Fatty Acids (SCFAs)-producting and 9 genera involved in Secondary Bile Acids (SBAs)-metabolism.**

| **Metabolites** | **Producers** | **References** |  |
| --- | --- | --- | --- |
| **SCFAs** | *Faecalibacterium prausnitzii, Roseburia intestinalis* | *Parada Venegas et al., 2019* |  |
|  | *Blautia faecis, Roseburia inulinivorans, Clostridium lavalense, Bacteroides uniformis* | *Takahashi K et al., 2016* |  |
|  | *Akkermansia muciniphila*, *Bacteroides* spp., *Bifidobacterium* spp., *Prevotella* spp., *Ruminococcus* spp. | *Louis et al., 2014, Rey et al., 2010* |  |
|  | *Blautia hydrogenotrophica*, *Clostridium* spp., *Streptococcus* spp. |  |  |
|  | *Bacteroides uniformis; Bacteroides vulgatus; Prevotella copri; Alistipes putredinis; Roseburia inulinivorans; Blautia obeum; Dialister invisus; Akkermansia muciniphila; Ruminococcus obeum; Veillonella parvula; Bacteroides eggerthii; Bacteroides fragilis* | *Louis et al., 2014, Rey et al., 2010; Nicole Reichardt et al.,2014;* |  |
|  |  |  |  |
|  |  |  |  |
| **SBAs** | *Faecalibacterium prausnitzii; Eubacterium rectale; Eubacterium ramulus; Eubacterium ventriosum; Eubacterium oxidoreducens; Roseburia cecicola; Roseburia intestinalis; Eubacterium hallii; Coprococcis eutactus; Clostridium butyricum; Roseburia faecis; Roseburia hominis; Clostridium composti; Anaerostipes hadrus; Coprococcus catus; Eubacterium biforme* | *Kumar Ganesan et al.,2018; Rebeca Martín et al.,2017; Louis P et al.,2009; Georgina L Hold et al.,2003; Louis et al.,2014; Cassir N et al.,2015; Shin Y et al.,2018* |  |
|  | *Lactobacillus* spp*., Bifidobacterium* spp*., Clostridium* spp*., Bacteroides* spp*.* | *Wahlström, A et al.,2016; Horáčková, Š et al.,2018* |  |
|  | *Bacteroides, Clostridium, Escherichia, Egghertella, Eubacterium,and Ruminococcus* | *Brown and Hazen,2018* |  |
|  | *Bacteroides, Eubacterium and Lactobacillus* | *Benson GM et al.,1998* |  |
|  | *Clostridium, Bifidobacterium, Enterococcus, Lactobacillus, Bacteroides* | *Dawson PA et al.,2015; Ridlon JM et al.,2014; Wahlstrom A et al,.2016; Klaassen CD et al.,2015; Ridlon JM et al.,2016* |  |
