# Supplementary material for: Comparison of Fecal Collection Methods on Variation in Gut Metagenomics and Untargeted Metabolomics
Source: mSphere. 2021 Sep 15;6(5):e00636-21. doi: 10.1128/mSphere.00636-21 (PMC8550109; doi:10.1128/mSphere.00636-21)
Supplement: TEXT S1 [file msphere.00636-21-s0001.pdf]

Document project information

Application number | Application date
CN201710429297.7 | 2017-06-08
Open (announcement) number | Open (announcement) date
CN107034141A | 2017-08-11
classification codes
C12N1/04(2006.01)I

【Applicant】
Shenzhen micro health gene technology co., LTD
【Inventor】
Zhenyu Yang, Wenkui Dai, Qian Zhou, et al.
【Agent】
Shenzhen micro health gene technology co., LTD
【Agency】
Beijing kuai wisdom intellectual property agency co., LTD
【Address】
No.1803, Jin Hecheng Investment Building, Wuhe Avenue, Bantian Street, Longgang District, Shenzhen City, Guangdong Province, China

【Claim】

Microlution is a relatively new stool collection kit, designed for the collection and preservation of the intestinal flora of fecal samples. Material components per every 1000 mL Microlution fixative include 400~600 mmol Tris-HC1, 3~7 mol NaCl, 50~150 mmo1 EDTA and/or EDTA-Na2, and 1~3 mol ethanol; the balance is water.

Protocol of Microlution

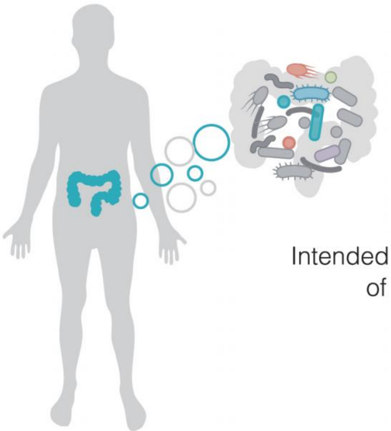

For In Vitro Diagnostic Use
Contains 1 collection kit

Intended for the collection and stabilization of microbial DNA from fecal sample.

Microlution

Sampling Instructions

4 Simple Steps to Sample

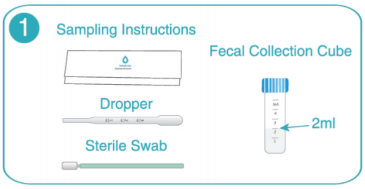

Get Ready:

- Check the package: Sampling Instructions, Dropper, Sterile Swab, Fecal Collection Tube;
- Fecal Collection Tube with 2ml liquid;
- Remove the lid from fecal collection tube, but keep it nearby;

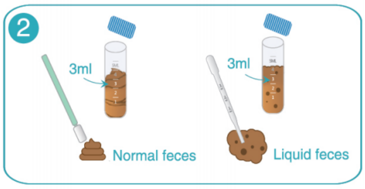

Sampling: (Collect from the middle of the feces)

- If normal feces, please collect with swab;
  If liquid feces, please collect with dropper;
- Collect enough samples to make sure the liquid level reaches the mark '3 ml';

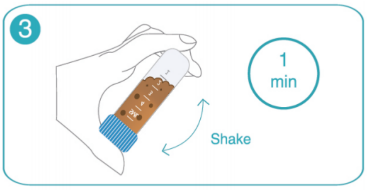

Shake:

- Tightly replace the lid;
- Shake the tube about 1 minute;

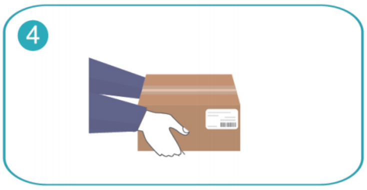

Send:

- Hand the tube to the lab staff
  OR
- Place the tube in a mailer; Drop the mailer in any mailbox;

Notices

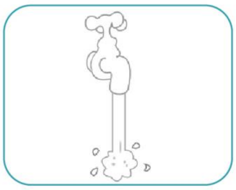

- Keep the reagents away from children;
- In case of contact with eyes or skin, please rinse with clear water;

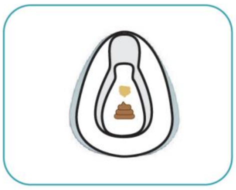

- Recommended disposable potty;
- Keep away from the surfaces of potty and avoid contamination with debris and urine;

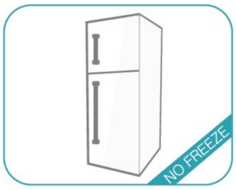

- Do not freeze;
- Place in room temperature or refrigerate with 4℃ (39.2°F)
